# An evolutionary compass for detecting signals of polygenic selection and mutational bias

**DOI:** 10.1002/evl3.97

**Published:** 2019-01-25

**Authors:** Lawrence H. Uricchio, Hugo C. Kitano, Alexander Gusev, Noah A. Zaitlen

**Affiliations:** ^1^ Department of Biology Stanford University Stanford CA; ^2^ Department of Computer Science Stanford University Stanford CA; ^3^ Dana Farber Cancer Institute Boston MA; ^4^ Department of Medicine University of California San Francisco CA; ^5^ Bioengineering and Therapeutic Sciences University of California San Francisco CA

**Keywords:** Complex traits, mutation bias, natural selection, polygenic selection

## Abstract

Selection and mutation shape the genetic variation underlying human traits, but the specific evolutionary mechanisms driving complex trait variation are largely unknown. We developed a statistical method that uses polarized genome‐wide association study (GWAS) summary statistics from a single population to detect signals of mutational bias and selection. We found evidence for nonneutral signals on variation underlying several traits (body mass index [BMI], schizophrenia, Crohn's disease, educational attainment, and height). We then used simulations that incorporate simultaneous negative and positive selection to show that these signals are consistent with mutational bias and shifts in the fitness‐phenotype relationship, but not stabilizing selection or mutational bias alone. We additionally replicate two of our top three signals (BMI and educational attainment) in an external cohort, and show that population stratification may have confounded GWAS summary statistics for height in the GIANT cohort. Our results provide a flexible and powerful framework for evolutionary analysis of complex phenotypes in humans and other species, and offer insights into the evolutionary mechanisms driving variation in human polygenic traits.

**Impact Summary**

Many traits are variable within human populations and are likely to have a substantial and complex genetic component. This implies that mutations that have a functional impact on complex human traits have arisen throughout our species' evolutionary history. However, it remains unclear how evolutionary processes such as natural selection may have acted to shape trait variation at the genetic and phenotypic level. Better understanding of the mechanisms driving trait variation could provide insights into our evolutionary past and help clarify why it has been so difficult to map the preponderance of causal variation for common heritable diseases.

In this study, we developed and applied methods for detecting signatures of mutation bias (i.e., the propensity of a new variant to be either trait‐increasing or trait‐decreasing) and natural selection acting on trait variation. We applied our approach to several heritable traits, and found evidence for both natural selection and mutation bias, including selection for decreased body mass index [BMI] and decreased risk for Crohn's disease and schizophrenia.

While our results are consistent with plausible evolutionary scenarios shaping a range of traits, it should be noted that the field of polygenic selection detection is still new, and current methods (including ours) rely on data from genome‐wide association studies (GWAS). The data produced by these studies may be vulnerable to certain cryptic biases, especially population stratification, which could induce false selection signals. We therefore repeated our analyses for the top three hits in a cohort that should be less susceptible to this problem—we found that two of our top three signals replicated (BMI and educational attainment), while height did not. Our results highlight both the promise and pitfalls of polygenic selection detection approaches, and suggest a need for further work disentangling stratification from selection.

Natural selection and mutation shape variation within and between populations, but the evolutionary mechanisms shaping causal variation for human traits remain largely unknown. Studies of selection in humans have often focused on classic selective sweeps (Sabeti et al. 2002, 2006; Voight et al. 2006; Hernandez et al. 2011; Enard et al. 2014), but other processes such as stabilizing selection (Gilad et al. 2006; Sanjak et al. 2018), polygenic adaptation (Turchin et al. 2012; Berg and Coop 2014), background selection (Charlesworth 1994; McVicker et al. 2009), negative selection (Boyko et al. 2008), and soft sweeps (Messer and Petrov 2013; Schrider and Kern 2017) may also play an important role in shaping human diversity. Methods to detect selection under these more complex models are needed if we are to fulfill the promise of genomics to explain the evolutionary mechanisms driving the distribution of heritable traits in human populations (Pritchard et al. 2010).

With the recent proliferation of paired genotype and phenotype data from large human cohorts, it is now feasible to test for polygenic selection on specific traits (Turchin et al. 2012; Berg and Coop 2014; Robinson et al. 2015; Yang et al. 2015; Field et al. 2016). While these studies have argued that polygenic selection is likely to be an important determinant of variation in traits such as height and skin pigmentation (Berg and Coop 2014), important questions remain about the evolutionary mechanisms that drive complex trait variation. In particular, most previous studies of selection on human complex traits have focused either on polygenic adaptation (Turchin et al. 2012; Berg and Coop 2014; Field et al. 2016; Berg et al. 2017; Edge and Coop 2018; Racimo et al. 2018) or stabilizing/negative selection (Yang et al. 2015; Zeng et al. 2018; Simons et al. 2018), and have not incorporated mutational bias (i.e., the propensity for new mutations to be preferentially trait‐increasing or preferentially trait‐decreasing). To detect polygenic adaptation, studies have relied on genotype data from multiple populations to probe the frequency and linkage properties of trait‐associated alleles as compared to a null based on genome‐wide single nucleotide polymorphisms (Turchin et al. 2012; Berg and Coop 2014; Racimo et al. 2018), or have used haplotype‐based statistics and dense sequence data from a single population (Field et al. 2016). Negative selection has been investigated by comparing empirical data to null models of the relationship between frequency and squared effect sizes (Schoech et al. 2017) or linkage disequilibrium and per‐single nucleotide polymorphism heritability (Gazal et al. 2017). These studies have argued that selection acts on many traits, and that both negative (Gazal et al. 2017; Schoech et al. 2017) and positive (Berg and Coop 2014; Berg et al. 2017) selection act on complex traits such as height and body mass index (BMI).

While each of these approaches has provided insights into the evolution of complex traits, a more comprehensive view of trait evolution will require methods that can account for pleiotropic selection and incorporate signals of both adaptive and deleterious selection processes simultaneously. Recent progress has been reported in accounting for higher dimensional trait spaces (Berg et al. 2017; Simons et al. 2018), but there is a need for models and inference tools integrating signatures of positive and negative selection on complex traits (Sanjak et al. 2018). Indeed, a natural way to model polygenic adaptation is to view stabilizing selection as a null process, with punctuated changes in the fittest trait value (herein called the “optimal trait value” or “trait optimum”) driving brief periods of adaptation (Barton 1986; Jain and Stephan 2017). Mutational bias may also be an important contributor to the evolutionary dynamics of complex traits (Charlesworth 2013), but has not been directly incorporated into recent empirical studies. If there is a bias in the direction of effect of de novo mutations, populations may carry less standing variation for alleles that alter the phenotype in one direction than the other, potentially altering the dynamics of future adaptation to changes in the trait optimum. Moreover, biases in mutation rate for selected traits may induce detectable patterns in the relationship between allele frequency and effect size, for example by driving an excess of trait‐increasing mutations among young (low‐frequency) alleles but not old alleles.

Here, we develop a powerful method for detecting differences between ancestral and derived allele effect sizes—which may be driven by selection or mutational bias—within a single population. We show that the polarization of GWAS summary statistics by their ancestral/derived state (which we refer to as an evolutionary compass) provides information about the evolutionary processes shaping trait variation. We propose a simple summary statistic of the relationship between effect sizes and allele frequency, and show that it is sensitive to both mutational bias and various models of selection. We apply our approach and find nonneutral signals that are consistent with selection and mutational bias in the genetic variation underlying BMI, educational attainment, Crohn's disease, schizophrenia, and height. We develop a model‐based inference procedure to disentangle mutational bias from selection, and show that both processes are necessary to explain the observed GWAS summary data. We then perform a replication study in the UK Biobank for our top three signals, and find that educational attainment and BMI replicate in this more homogeneous cohort, while height does not. We discuss implications of our findings for human evolutionary history and GWAS of biomedically relevant traits.

## Results

### AN “EVOLUTIONARY COMPASS” FOR GWAS

**Figure 1:**
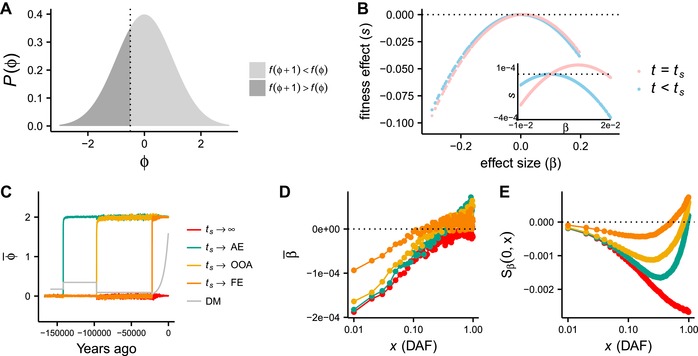
Panels A‐B are schematics of the trait model, while C‐E show simulation results. A: fitness impact of a β=1 mutation, assuming a symmetric fitness function. At equilibrium, the trait distribution P(φ) is symmetric about the optimal value of the phenotype, $\varphi_{o}=0$. The dashed line at $\varphi =-\frac{1}{2}$ indicates the dividing line between individuals with increased fitness *f* after a β=1 mutation (f(φ+1)>f(φ)) from those with decreased fitness (f(φ+1)<f(φ)). B: schematic of the relationship between effect size and fitness effect. At time $t=t_{s}$, the optimal trait value $\varphi_{o}$ increases, and trait‐decreasing alleles have decreased fitness while trait‐increasing alleles have increased fitness. Still, only trait‐increasing alleles of small effect are on average fitness‐increasing (inset). C: Mean trait value $\bar{\varphi }$ as a function of time for four simulated trait models, differentiated by the time of a shift in selection pressure. The simulated European demographic model is plotted in the background (not to scale) D: $\bar{\beta }$ as a function of derived allele frequency (DAF) for each model simulated in C. Points represent the mean value of β computed over 100 independent simulations E: $S_{\beta }\left(0,x\right)$ as a function of DAF for each model plotted in C. D and E represent the mean over 100 independent simulations. (Abbreviations: *AE*: ancestral expansion, *OOA*: out‐of‐Africa, *FE*: founding of Europe, *DM*: demographic model).

Associations between genotypes and complex traits are usually reported with respect to a reference allele that is arbitrarily chosen, which can obscure the direction of effect of new mutations. When selection acts on traits, it favors the reproductive success or survival of individuals with particular trait values, implying that the fitness effect of new mutations may depend on both the sign and magnitude of their impact on a selected trait. Therefore, we choose ancestral alleles as the reference state and explore the relationship between derived allele effect sizes and derived allele frequency, which we encode in a βDAF plot (i.e., a plot encoding the relationship between the mean value of effect sizes β estimated in a GWAS ($\bar{\beta }$) and derived allele frequency (DAF); Fig. 1). Note that we use estimated effect sizes from all alleles to compute $\bar{\beta }$, regardless of their significance.

A βDAF plot contains information about mutational bias and selection on the trait of interest. In the null case of a neutrally evolving trait for which trait‐increasing and trait‐decreasing mutations are equally likely, the βDAF curve will be flat will have expectation $\bar{\beta }=0$ in all derived allele frequency bins. This is because in a neutral model, the probability with which an allele segregates at frequency *x* does not depend on effect size β (Fig. S2). A mutational bias toward trait‐increasing or trait‐decreasing alleles in the absence of selection on the trait will shift the mean value of β in the direction of the bias, but will not induce $\bar{\beta }$ to depend on frequency (Fig. S2).

While many evolutionary processes will induce patterns in the βDAF plot (including directional selection, which we explore in the Supporting Information with simulations and analytical calculations in Figs. S1–S3), we next consider an example of stabilizing selection with shifts in the optimal trait value for illustrative purposes. Stabilizing selection is typically parameterized by several parameters, but here we focus on the optimal phenotype value, $\varphi_{o}$, which represents the value of the phenotype that confers the highest fitness. Applying a classic stabilizing selection model, we suppose that fitness is controlled by a Gaussian function centered at $\varphi_{o}$ (Robertson 1956; Barton 1986; Simons et al. 2018) (Fig. 1A). In addition, we suppose that trait‐increasing mutations may be more or less likely than trait‐decreasing mutations, which we capture with the parameter δ (defined as the proportion of trait‐altering de novo mutations that increase trait values), and that $\varphi_{o}$ can change, inducing a brief period of adaptation in which trait values within the population equilibrate to a new optimal value (Fig. 1B).

We performed forward simulations of complex traits under this model and explored how various evolutionary parameters affect the properties of a βDAF plot. Although we consider a very large range of possible parameter combinations when performing statistical inference in later sections, here we focused on four models, including one model of stabilizing selection in the absence of shifts in the optimal trait value, and three models that varied the timing of a shift ($t_{s}$) in the optimal trait value $\varphi_{o}$ (Fig. 1C). All of the models included a bias in mutation rate toward trait‐decreasing alleles (δ=0.4) and a European demographic model that was fit to patterns of European genomic diversity (Gravel et al. 2011). For models that include a shift in the optimal trait value, we considered a change (denoted Δφ) equal to two SDs of the trait distribution. For reference, this would correspond to approximately a 5 inch change in mean human height (Fryar et al. 2016). The remaining model parameters for the simulations in Figure 1 are given in the Supporting Information (page 26). To compute the mean effect size $\bar{\beta }$ (Fig. 1D), we grouped alleles into 1% frequency bins and computed the mean effect size across all derived alleles within the bin.

When there is a bias toward trait‐decreasing alleles and stabilizing selection acts on the trait, alleles at low frequencies have strongly negative effect sizes, which generate a positive correlation between effect size and frequency (Fig. 1D, red curves). When the optimal value of the phenotype increases in response to an environmental shift, the relationship between effect size and selection coefficient also transiently changes (Fig. 1B), driving some alleles with beneficial effects to higher frequencies (Fig. 1D, yellow, green, and orange curves). This effect transiently changes the relationship between allele frequency and effect size by promoting trait‐increasing alleles to higher frequencies, generally increasing $\bar{\beta }$ at all frequencies. Although $\bar{\beta }$ is always negative at low frequencies for the particular parameter combinations we investigated here, at high frequencies, $\bar{\beta }$ can become positive due to the preferential increase in frequency of trait‐increasing alleles. These effects decay as the time since the shift event increases (Fig. 1D). The slow decay indicates that such patterns might be detectable in trait data for tens of thousands of years, with the time‐scale for detection depending on the model parameters and the precision of effect size estimates in GWAS summary data.

### A STATISTICAL TEST FOR POLYGENIC SELECTION AND MUTATIONAL BIAS ON ORIENTED GWAS

Selection and mutational bias change the relationship between $\bar{\beta }$ and DAF (Fig. 1; Figs. S1–S3). We desire a simple statistic that will differ from zero when selection and/or mutational bias act. We first consider the integral of the area under a βDAF plot, noting that while other statistics are also likely to be informative (Yang et al. 2015), not all choices will be robust to ancestral state uncertainty (see Supporting Information). We approximate this integral with the sum ($S_{\beta }$) over $\bar{\beta }$ for derived allele frequency bins of some width $y_{w}$.

(1) $$S_{\beta }\left(f_{i},f_{j}\right)=\sum_{y\in Y_{ij}}\bar{\beta }\left(y\right).$$

As in Fig. 1, we group alleles into frequency bins of width $y_{w}$ such that the set *Y* is given by the sequence of frequency tuples $Y=\langle \left(0,y_{w}\right),\left(y_{w},2y_{w}\right),\cdots \left(1-y_{w},1\right)\rangle$. $Y_{ij}$ refers to the subsequence of *Y* that includes all elements indexed between *i* and *j*, and $f_{i}$ is the lower frequency in the *i*‐th tuple while $f_{j}$ is the higher frequency in the *j*‐th tuple. We choose $y_{w}=0.01$ such that there are a large number of alleles within each bin.

The $S_{\beta }$ statistic is sensitive to both mutational bias and selection, although these two distinct evolutionary processes drive distinct patterns in a βDAF plot. When mutational bias acts on new mutations in the absence of selection on the trait, there is no expectation of a relationship between the magnitude of effects and allele frequency, and hence the expected value of β is the same in all allele frequency bins (Fig. S2). This means that the expected value of $S_{\beta }$ is simply equal to the mean effect size of new mutations multiplied by the number of bins, and that the sign of $S_{\beta }$ is equal to the sign of the bias in mutational effects.

In the absence of mutational bias, $S_{\beta }$ is sensitive to some (but not all) selection models. When a trait is under long‐term stabilizing selection with no change in the optimal phenotype and no mutational bias, trait‐increasing alleles are equally deleterious and equally likely to occur as trait‐decreasing alleles, meaning that the expectation of $S_{\beta }$ is 0 (which concords exactly with negative selection models—Fig. S3, purple curves). However, any bias in mutation rate toward trait‐increasing or ‐decreasing alleles will drive $S_{\beta }$ to have non‐zero expectation (Fig. S3). In contrast to the neutral case with mutational bias, selection in conjunction with mutational bias causes the mean value of β to be greater in magnitude in bins of low derived allele frequency than high allele frequency, since selection will tend to constrain alleles with the largest effects to the lowest frequencies (Fig. 1C–E, red curves; Fig. S3).

The most interesting patterns emerge when stabilizing selection acts on a trait in conjunction with shifts in the fitness optimum. When stabilizing selection acts on a trait under along with mutational bias (δ=0.4) and no shift in the optimum, $S_{\beta }\left(0,x\right)$ is negative at all *x*, and decreases as a function of frequency (red curve, Fig. 1E; we show in Fig. S3 and that this same pattern holds under a well‐studied directional selection model for complex traits (Eyre‐Walker 2010)). When shifts in the optimum toward higher trait values occur, higher frequency variants have mean positive effect sizes, causing $S_{\beta }\left(0,x\right)$ to be non‐monotonic and $S_{\beta }\left(0,1\right)$ to potentially have positive sign. As time elapses since the shift, the high frequency trait‐increasing alleles will tend to drift to the boundary and fix or be lost, causing this signal to gradually disappear (Fig. 1E). Note that mutational bias in the absence of selection can generate a non‐zero $S_{\beta }$—however, mutational bias alone induces $S_{\beta }\left(0,x\right)$ to increase in magnitude monotonically and linearly in *x*, a pattern that is not expected for selection (Fig. S2 and Supporting Information). We use model‐based analyses to tease apart mutational bias and selection effects in later sections.

### A PERMUTATION PROCEDURE TO GENERATE A NULL DISTRIBUTION

While we have noted that $S_{\beta }$ has an expected value of 0 under the neutral null without mutational bias, the variance of $S_{\beta }$ depends on linkage between causal alleles and noncausals, since noncausal alleles will have non‐zero estimated effect sizes when they are in Linkage disequilibrium (LD) with a causal allele. To control for potential confounding by LD, we developed a simple permutation‐based procedure for computing the null‐distribution of frequency‐effect size relationships in GWAS summary statistics under a neutral evolutionary model. We first polarize all alleles such that the derived allele is the causal allele. Then, for each of 1703 previously identified approximately independent linkage blocks (Berisa and Pickrell 2016), we select a random sign (positive or negative with equal probability), and multiply all the effect sizes in the LD block by this sign. We then recompute the test statistic of interest, such as the correlation between frequency and MAF, on the randomized data. This generates a null distribution for the test statistic that conservatively accounts for the linkage between inferred effect sizes, and maintains the frequency spectrum and marginal distribution of the magnitude of inferred effect sizes.

### APPLICATION TO GWAS SUMMARY DATA

**Figure 2:**
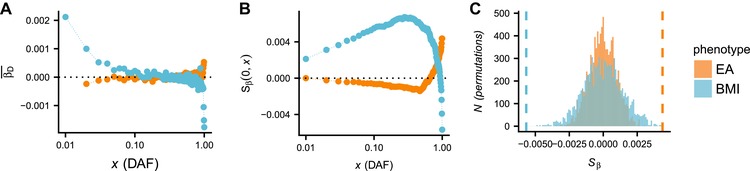
A: $\bar{\beta }$ as a function of DAF for BMI and educational attainment (EA). B: $S_{\beta }\left(0,x\right)$ for the same data. C: neutral null distribution of $S_{\beta }\left(0,1\right)$ obtained by permutations. The vertical dashed line indicates the observed value of $S_{\beta }\left(0,1\right)$ in the GWAS summary data.

We applied $S_{\beta }$ within our framework to GWAS summary data for BMI, height (Wood et al. 2014), and educational attainment (Okbay et al. 2016) to assess its power for detecting selection, given that previous studies have suggested that these phenotypes may be under selection (Turchin et al. 2012; Berg and Coop 2014; Robinson et al. 2015; Field et al. 2016; Racimo et al. 2018). We observe that effect sizes are correlated with frequency, and that $S_{\beta }$ is a non‐monotonic function of frequency (Fig. 2), consistent with selection and mutational bias (Fig. 1E; note that the large spike in both BMI and height at high frequency can be explained by ancestral uncertainty, Fig. S7). We reject the neutral null for all three traits ($P<5\times 10^{-4}$; Fig. 2 and Table 1). We then applied our method to six additional phenotypes, which we selected to span a wide range of phenotypes that we hypothesized might be targets of selection, including body size (Wood et al. 2014), psychiatric conditions (CDG Psychiatric Genomics Consortium 2013), immune‐related traits (Franke et al. 2010), reproductive traits (Day et al. 2015), and cardiovascular traits (Willer et al. 2013). We find an additional nonneutral signal for Crohn's disease, and a marginally significant signal for schizophrenia that narrowly missed a multiple testing correction (Table 1; Figs. S8–S16). When including only common variants in the test, we find that seven of nine phenotypes have *P*‐values under 0.1, suggesting strong enrichment for selected traits among the test set despite failure for some of the tests to exceed a multiple testing correction (binomial *P*‐value $3.0\times 10^{-6}$).

**Table 1:** *P*‐values corresponding to GWAS summary statistics for nine phenotypes that we hypothesized may be under selection. Values in the first column include all alleles, while the second and third columns correspond to tests including only alleles with MAF > 1% and MAF > 5%, respectively. The UK Biobank tests were performed on all alleles above 1% in frequency

Phenotype	$S_{\beta }\left(0,1\right)$ *P*‐value	$S_{\beta }\left(0.01,0.99\right)$ *P*‐value	$S_{\beta }\left(0.05,0.95\right)$ *P*‐value	UKBB replication
Height	<0.0005**	<0.0005**	<0.0005**	0.416
BMI	<0.0005**	<0.0005**	<0.0005**	0.0095
Education	<0.0005**	<0.0005**	<0.0005**	<0.0005
WHR‐BMI	0.566	0.8325	0.341	NA
GLL	0.4655	0.434	0.5645	NA
Crohn's disease	0.0025**	0.0075*	0.018*	NA
Menopause onset	0.1585	0.46	0.0475*	NA
Depression	0.3915	0.01*	0.0305*	NA
Schizophrenia	0.0085*	0.0035**	0.0625	NA

^*^Tests that pass a multiple testing correction (P<0.005).

^**^Tests that were marginally significant (P<0.05).

### EVOLUTIONARY MODELS FOR SELECTION ON HUMAN TRAITS

While our results show that $S_{\beta }$ has a strong nonneutral signature for five of the nine phenotypes, we sought to further understand the evolutionary models that could explain these signals. Purifying selection alone seems an unlikely candidate for most of the phenotypes, because the sign of $S_{\beta }$ is always the same as the sign of $\bar{\beta }$ under a purifying selection model (Fig. S3 and Supporting Information), a pattern violated by height, BMI, Crohn's, and educational attainment. Models of directional selection for increased or decreased phenotype values in the absence of mutational bias also share this pattern, as do models of mutational bias in the absence of selection (Figs. S1 and S2; Supporting Information).

**Figure 3:**
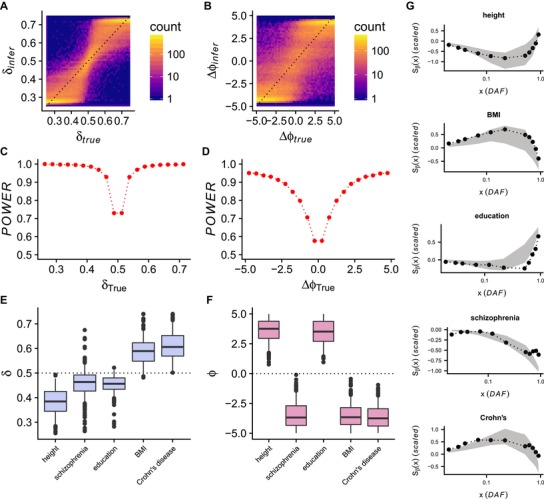
A‐B: Inferred mutation bias (A) and selection shift (B) parameters as a function of true parameter values for our rejection sampling method. C‐D: Power of our rejection sampling method to correctly identify the direction of mutation bias (C) and shift in optimal phenotype value (D), as a function of the true parameter value. E‐F: Inferred approximate posterior distributions for five phenotypes that were identified as non‐neutral. G: Out‐of‐sample simulations using parameters inferred in E‐F, plotted with the data used to fit each model. Gray envelopes represent the middle 50% of simulation replicates, while the black points and curves show the observed data for each phenotype.

We hypothesized that these signals could be explained by a model of stabilizing selection, mutational bias, and shifts in the trait value conferring optimal fitness. We developed an evolutionary inference procedure based on rejection sampling (Tavaré et al. 1997) to infer the parameters that best fit the relationship between frequency and effect size that we observe in the data (see Supporting Information). Briefly, we calculate $S_{\beta }\left(0.01,x\right)$ (scaled by $S_{\beta }\left(0.01,0.99\right)$) for a range of derived allele frequencies *x*, which we then use as summary statistics for rejection sampling. We remove the lowest frequency variants (i.e., those with x<0.01) for the purpose of this inference to avoid the potential impact of rare variant stratification on our results. We validated our method with extensive simulations, and found that it is a noisy estimator of the magnitude of δ and Δφ (Fig. 3A and B—recall that δ corresponds to the proportion of new mutations that are trait‐increasing), but has excellent power to estimate the direction of both mutational bias and shift in optimal trait value (Fig. 3C and D). Other parameters of the model (including heritability, polygenicity, effect size distribution, and the time of the shift in the fitness landscape; see Supporting Information) were inferred with low accuracy as indicated by only modest correlations between inferred and true parameter values, indicating that the summary statistics we use contain little information about these parameters. Inferred distributions of these parameters therefore are not reported.

We find strong posterior support for a shift in optimal trait value (Δφ, measured in units of SDs of the population trait distribution) for all five phenotypes, as well as strong signals of mutational bias (δ). Data for height and educational attainment supported a shift toward increased trait values and a mutational bias toward mutations that decrease the phenotype, while Crohn's disease and BMI supported shifts toward lower trait values and mutational bias toward trait‐increasing alleles (i.e., risk‐increasing for Crohn's). Schizophrenia data supported a shift toward lower risk, and a bias toward protective mutations, although a substantial minority of parameter estimates supported no mutational bias or a bias in the opposite direction (Fig. 3E). To further validate these findings, we resampled from the inferred parameter distributions and performed an independent set of forward simulations. We find that the summary statistics computed on these out‐of‐sample simulations (which were not used to fit the data) match the trends observed in our data, confirming that the modeling framework is capable of recapitulating the signals we observe in the data (Fig. 3G).

### POPULATION STRATIFICATION

Although $S_{\beta }$ is not sensitive to confounding by ancestral uncertainty, we were concerned that these signals could potentially be explained by other confounders, such as uncorrected population stratification. Although recent studies have suggested that methods to account for stratification in GWAS often over‐correct (Field et al. 2016; Bulik‐Sullivan et al. 2015), rare variant stratification remains especially difficult to account for in GWAS (Mathieson and McVean 2012). We recomputed $S_{\beta }$ on alleles with MAF >1% and MAF >5%, and found that the signals are robust within common alleles alone, suggesting that population structure is unlikely to confound our estimates (Figs. S8–S16 and Table 1). Additionally, we recomputed $S_{\beta }$ on GWAS from the UK Biobank (UKBB) for our top three signals, a replication cohort that employed strict population structure control in a more homogeneous group of samples. We replicate the signals for both educational attainment ($P<5\times 10^{-4}$) and BMI (P=0.0095), noting that the educational attainment data in our two datasets contain some overlapping samples and hence are only partially independent and the BMI signal is somewhat weaker in UKBB than GIANT (Supporting Information and Fig. S17). Interestingly, height was not replicated. We also applied two previous approaches that detected signals of selection acting on height when analyzing the GIANT summary statistics and found that neither replicated in the UKBB (Turchin et al. 2012; Yang et al. 2015; Fig. S6). These results are consistent with either overcorrection of structure within the UKBB or undercorrection in the GIANT data, although the latter seems more likely based on other recent studies (Berg et al. 2018; Sohail et al. 2018—see Discussion).

## Discussion

Many studies have suggested that selection shapes human genetic variation (e.g., Fay et al. 2001), and recent work has suggested that selection on complex traits may be a substantial driver of human adaptation (Hernandez et al. 2011). Here, we developed a novel empirical framework and a model‐based rejection sampling approach for detecting polygenic selection and mutational bias that can be applied to GWAS summary data for a single population. We call this approach an “evolutionary compass,” because orienting alleles by their ancestral/derived status within our framework provides insight into the evolutionary processes shaping complex traits. We applied this evolutionary compass to GWAS summary data for nine phenotypes, and showed that five of them (educational attainment, height, Crohn's disease, BMI, and schizophrenia) are consistent with a model of selection and mutation bias in shaping trait variation. Interestingly, among the top three signals that were uncovered with our method, height did not replicate in a more homogeneous cohort, while both BMI and educational attainment were replicated.

If selection acts on biomedically relevant complex traits such as Crohn's disease and schizophrenia, there are important implications for the future of both medical and evolutionary genomics. In medical genomics, an ongoing debate about the genomic architecture of complex diseases is at the forefront of the field (Manolio et al. 2009). When strong selection acts on complex traits, it can elevate the role of rare alleles in driving trait variance (Lohmueller 2014). If rare alleles contribute a larger fraction of the genetic variance than is expected under neutral models, then very large GWAS that use only array‐based genotyping information are very unlikely to be able to capture these signals, and sequence‐based studies and powerful rare variant approaches that are robust to evolutionary forces (including those not investigated here, such as partial recessivity) will be needed (Uricchio et al. 2016; Hernandez et al. 2017; Sanjak et al. 2017). Moreover, recent work has suggested that the over‐representation of Europeans in GWAS has limited the effectiveness of estimating polygenic risk scores in other human populations (Martin et al. 2017). This is problematic for the transfer of genomic research into the clinic, where precision medicine initiatives relying on personal genetic information will be most successful if genetic risk can be accurately predicted in diverse populations. While this inability to predict across populations could be driven by neutral demographic forces, if selection has driven numerous phenotypes to acclimate to local environmental conditions in ancestral human populations worldwide it could exacerbate this problem dramatically.

In the field of human evolutionary genomics, most studies have agreed that the impact of selection is widespread on the human genome, but the evolutionary mechanisms that drive genetic and phenotypic diversity have been widely debated (Hernandez et al. 2011; Enard et al. 2014; Schrider and Kern 2017). In our study, we showed that GWAS summary statistics in Europeans for BMI and Crohn's disease are consistent with a bias in mutation rate toward trait‐increasing alleles, and a shift to a lower optimal value of the trait, while educational attainment is consistent with a mutational bias toward trait‐increasing alleles and a shift toward higher values of the trait optimum. The signal for schizophrenia is consistent with an ancestral shift toward a lower optimum and a stabilizing selection, with or without a mutational bias. It should be noted that the model we used to fit these data assumed no more than one shift in the optimal phenotype value, whereas this quantity is likely to vary continuously with environmental conditions for some traits. Models that additionally account for sexual dimorphism (Stulp and Barrett 2016), higher dimensional trait spaces (Simons et al. 2018), and evolutionary history of multiple populations (Berg and Coop 2014; Racimo et al. 2018) may be required to better understand the generality of these results across human populations and traits.

Neanderthal introgression into modern humans has played an important role in shaping traits in non‐African populations (Wall et al. 2013; McCoy et al. 2017). Given that Neanderthal alleles may contribute disproportionately to the genetic variance in some traits (Simonti et al. 2016) and that some high frequency trait‐associated alleles have Neanderthal origins (Dannemann and Kelso 2017; Prüfer et al. 2017), we hypothesized that Neanderthal alleles for traits under selection might show distinct patterns from modern human alleles. Although Neanderthal alleles do not share a common demographic history with modern human alleles, under the neutral null hypothesis we do not expect Neanderthal alleles to have an increasing or decreasing frequency and effect‐size relationship, or to have a distribution that differs substantially from modern human alleles. We, therefore, computed $S_{\beta }$ on alleles that have a Neanderthal origin (see Supporting Information). Alleles for height and depression show strikingly different patterns than alleles with modern human origins (Fig. S18). The results for height can be explained by selection to promote Neanderthal height‐increasing alleles to high frequency, either along the Neanderthal lineage predating human introgression, or after admixture with human populations. In contrast, our results for depression risk are consistent with an excess of depression risk from Neanderthals (Simonti et al. 2016), and selection preferentially driving large effect alleles to low frequency. We note that while Neanderthal alleles are not subject to the same biases in ancestral state uncertainty as modern human alleles, inferences of selection could still be biased by population stratification.

Inference of selection on complex traits is vulnerable to several possible confounders, including population stratification and pleiotropic selection on off‐target phenotypes (Novembre and Barton 2018). Although theory suggests that stratification should be straightforward to detect and correct at high frequency variants in large samples (Patterson et al. 2006), an uncorrected bias in the inferred β values due to population structure can make our test (as well most others, such as Berg and Coop 2014; Yang et al. 2015; Field et al. 2016; Berg et al. 2017; Racimo et al. 2018) anti‐conservative. We used a series of experiments to show that population structure is unlikely to bias the majority of our results, including showing that the signal is robust to the exclusion of rare alleles and performing a replication study in an external cohort. However, one of our strongest signals (height) did not replicate the UK Biobank, while two other signals of selection suggested by earlier height studies also did not replicate in the UK Biobank (Turchin et al. 2012; Yang et al. 2015). The nonreplication of height is in concordance with other recent studies finding reduced evidence for selection on height in the UK Biobank cohort applying different methods (Berg et al. 2018; Sohail et al. 2018). The most conservative interpretation of the nonreplication of height is to suppose that some of the signals we and others observed in the GIANT cohort are driven by population stratification, and the UK Biobank analysis correctly removes this spurious contamination. Further research is needed to better disentangle stratification and selection, and caution in interpreting the results of polygenic selection tests is warranted while this field develops, even in more homogeneous cohorts such as the UK Biobank (Novembre and Barton 2018).

Pleiotropy can also induce biases in complex trait selection detection. Selection on a trait that has correlated effect sizes with another trait could result in false positives, in which the neutral trait is spuriously identified. This is a general limitation of most complex trait selection methods (but see Berg et al. 2017; Simons et al. 2018). This phenomenon is clearly highlighted by our results on educational attainment, a phenotype that had no meaning until recent historical times. While it might be tempting to identify a cognition‐related phenotype as the target of selection, it is possible that any trait with a cryptic shared genetic basis and correlated effect sizes could be the target, and that the timing of any such shift to larger trait values could have predated the human migration out‐of‐Africa. Thus, we cannot rule out a role for correlated phenotypes in driving these signals, and our results do not imply differences in phenotypes or polygenic scores between Europeans and any other group. More work on disentangling selection targets with a common genomic basis will be needed as the field progresses.

Among the nine traits that we tested, we found that four had strong non‐neutral signals, and six of the nine had marginal evidence for nonneutrality (we have conservatively removed height, given its nonreplication in the UK Biobank). However, this does not imply that the others are not subject to selection or mutation bias. The power of our test depends on the strength of selection, the polygenicity of the trait, the heritability of the trait, the mutational bias, and the amount of ancestral uncertainty, in addition to the sample size of the GWAS. If a trait is under strong stabilizing selection, but the mutation rate of trait‐increasing and ‐decreasing alleles is exactly equal, then our test has no power. Because we rely on the polarization of alleles by ancestral state, increased uncertainty in ancestral state will also decrease our power (see Supporting Methods). Moreover, if selection is weak, a small number of causal alleles drive variance in the trait, or the trait is only weakly heritable, power is greatly diminished. However, increased sample sizes in GWAS will increase power, because the variance of effect size estimates for even weak effect causal alleles decreases with sample size. In addition to working directly on GWAS summary statistics from a single population, one strength of our permutation‐based approach is that other informative statistics, such as the absolute value of the deviation between ancestral and derived effect sizes, could also easily be applied, and may have higher power. In future studies, it will be advantageous to compare various summary statistics and to apply our approach to species undergoing rapid environmental changes.

## Materials and Methods

### SOFTWARE

We wrote software in Python to implement the statistical test described herein, and we developed a custom simulator of demography and selection. The code to run our statistical test is freely available online (https://github.com/uricchio/PASTEL). The simulations (and their validation) are described in the Supporting Information.

Associate Editor: S. Wright

## Supporting information


**Figure S1**. A: When selection does not prefer either trait‐increasing or trait‐decreasing alleles, and mutation bias does not act on a trait, then trait‐increasing (pink) and ‐decreasing (blue) alleles are expected to have identical frequency spectra and equal mean β values within each frequency bin.
**Figure S2**. The relationship between β and DAF when only mutational bias acts on the trait (i.e., there is no selection).
**Figure S3**. A: A βDAF plot for various values of mutational bias (δ) towards trait decreasing alleles for traits under selection.
**Figure S4**. A comparison of simulated frequency spectra for a complex demographic/selection model for our Wright‐Fisher simulator (black) and SFS_CODE (red).
**Figure S5**. Conservative False Positive Rate estimation for detection of mutation rate bias and shifts in optimal phenotype value.
**Figure S6**. Correlation between mean effect size and allele frequency (A) and correlation the difference in frequency between northern and southern Europe for the height increasing allele and p‐value rank (B) in the GIANT study (gold) and the UK Biobank (gray).
**Figure S7**. The impact of ancestral uncertainty on the observed value of mean β as a function of derived allele count *x*.
**Figure S8**. $S_{\beta }$ for height.
**Figure S9**. $S_{\beta }$ for BMI.
**Figure S10**. $S_{\beta }$ for educational attainment.
**Figure S11**. $S_{\beta }$ for Crohn's disease.
**Figure S12**. $S_{\beta }$ for schizophrenia.
**Figure S13**. $S_{\beta }$ for global lipid levels.
**Figure S14**. $S_{\beta }$ for menopause onset.
**Figure S15**. $S_{\beta }$ for major depression.
**Figure S16**. $S_{\beta }$ for waist‐hip ratio adjusted for BMI.
**Figure S17**. $S_{\beta }$ for BMI (A‐C), height (D‐F), and educational attainment (G‐I) in the UK Biobank.
**Figure S18**. Signals of putative polygenic selection and mutation bias on Neanderthal alleles.
**Figure S19**. A‐C: Simulations with the same parameters as Fig. 1C‐E, but with the shift in optimal phenotype of Δφ=2 occurring linearly over 100 generations (2500 years), rather than instantaneously in a single generation.

## Data Availability

We used publicly available GWAS summary data from each of the original studies of the phenotypes that we analyzed, including height, BMI, and BMI‐WHR (Wood et al. 2014), schizophrenia and major depression (CDG Psychiatric Genomics Consortium 2013), Crohn's disease (Franke et al. 2010), menopause onset (Day et al. 2015), educational attainment (Okbay et al. 2016), and global lipid levels (Willer et al. 2013). Post‐processed data files (which map individual alleles to linkage blocks in the human genome) from each of these studies are available upon request, or could be generated by downloading the original GWAS summary data and mapping the alleles using the linkage data (Berisa and Pickrell 2016). The UK Biobank summary data that we use in our replication study were obtained online and are also freely available (https://data.broadinstitute.org/alkesgroup/UKBB/).
