# Supplementary material for: An evolutionary compass for detecting signals of polygenic selection and mutational bias
Source: Evol Lett. 2019 Jan 25;3(1):69–79. doi: 10.1002/evl3.97 (PMC6369964; doi:10.1002/evl3.97)
Supplement: Supplementary file 1 — Figure S1. A: When selection does not prefer either trait‐increasing or trait‐decreasing alleles, and mutation bias does not act on a trait, then trait‐increasing (pink) and ‐decreasing (blue) alleles are expected to have identical frequency spectra and equal mean β values within each frequency bin. Figure S2. The relationship between β and DAF when only mutational bias acts on the trait (i.e., there is no selection). Figure S3. A: A βDAF plot for various values of mutational bias (δ) towards trait decreasing alleles for traits under selection. Figure S4. A comparison of simulated frequency spectra for a complex demographic/selection model for our Wright‐Fisher simulator (black) and SFS_CODE (red). Figure S5. Conservative False Positive Rate estimation for detection of mutation rate bias and shifts in optimal phenotype value. Figure S6. Correlation between mean effect size and allele frequency (A) and correlation the difference in frequency between northern and southern Europe for the height increasing allele and p‐value rank (B) in the GIANT study (gold) and the UK Biobank (gray). Figure S7. The impact of ancestral uncertainty on the observed value of mean β as a function of derived allele count x. Figure S8. Sβ for height. Figure S9. Sβ for BMI. Figure S10. Sβ for educational attainment. Figure S11. Sβ for Crohn's disease. Figure S12. Sβ for schizophrenia. Figure S13. Sβ for global lipid levels. Figure S14. Sβ for menopause onset. Figure S15. Sβ for major depression. Figure S16. Sβ for waist‐hip ratio adjusted for BMI. Figure S17. Sβ for BMI (A‐C), height (D‐F), and educational attainment (G‐I) in the UK Biobank. Figure S18. Signals of putative polygenic selection and mutation bias on Neanderthal alleles. Figure S19. A‐C: Simulations with the same parameters as Fig. 1C‐E, but with the shift in optimal phenotype of Δφ=2 occurring linearly over 100 generations (2500 years), rather than instantaneously in a single generation. [file EVL3-3-69-s001.pdf]

Supplemental Information for "An evolutionary compass for detecting signals of polygenic selection and mutational bias"

Lawrence H. Uricchio,[1*†] Hugo C. Kitano,[2*]
Alexander Gusev,[3‡] Noah A. Zaitlen[4,5‡†]

Departments of [1]Biology and [2]Computer Science, Stanford University, Stanford, CA; [3]Dana Farber Cancer Institute, Boston, MA; Departments of [4]Medicine and [5]Bioengineering and Therapeutic Sciences, University of California, San Francisco, CA

[†]To whom correspondence should be addressed: uricchil@gmail.com, noah.zaitlen@ucsf.edu

[*]These authors contributed equally to this work
[‡]These authors contributed equally to this work

An evolutionary compass to detect selection and mutational bias

Selection on quantitative traits is known to induce dependency between mean squared effect sizes and derived allele frequency (DAF) (Eyre-Walker, 2010; Uricchio et al., 2016), but the relationship between effect sizes $\bar{\beta}$ and DAF under selection models has not been widely explored. Under some models, for example when mutation rates for trait increasing and decreasing alleles are symmetric and the fitness cost of a mutation does not depend on the direction of its effect, selection will not induce a relationship between effect size and allele frequency. However, other models, such as those in which selection preferentially drives trait-increasing alleles to high frequency with selection pressure that increases as a function of β, may drive such a correlation (*e.g.*, Fig. S1A-C), which could potentially be detected in GWAS summary statistics data. We hypothesized that a better understanding of this relationship between β and DAF could provide new insights into the evolution of complex traits.

Models with symmetric mutation rates have often been applied to quantitative traits, but there is no *a priori* reason to suppose that this condition is met in natural populations. Indeed, it is possible that there are an unequal number of fixed bi-allelic sites genome-wide that can increase or decrease a phenotype relative to its current value, which naturally will change the relative rate of trait-increasing as compared to trait-decreasing alleles. For example, if past selection events have driven the phenotype to ever larger values, we might expect that the majority of trait-increasing alleles have already been fixed by positive selection in the evolutionary past, and that further recurrent mutations at these fixed sites would therefore decrease the phenotype.

Temporal variation in the optimal value of selected traits may also be an important determinant of the evolutionary dynamics of complex traits, as such changes may be a mechanism for polygenic adaptation (Jain and Stephan, 2017). When a population's environment is altered, perhaps by migration, a change in climate, or the elimination/introduction of competing species, it is likely that selection pressures on phenotypes will also change. If the population persists long enough in this new environment it is expected that the phenotype mean will approach the new phenotype optimum, and the population will again be at equilibrium. In the intervening time, while the population is out of equilibrium, allele frequencies will shift as a function of their effect on the phenotype. Our goal is to capture selection's impact on the causal variation during this out-of-equilibrium time period.

In this project, we sought to capture how mutational bias and selection pressure would affect the relationship between $\bar{\beta}$ and allele frequency, and to use this information in designing a statistical test to detect mutational bias and selection. In the following sections, we use analytical theory and simulations to build intuition about the relationship between these evolutionary processes and patterns that could be observed in GWAS summary statistic data.

Building intuition with the βDAF plot and S_β

To build intuition about how evolutionary parameters affect the relationship between β and DAF we performed analytical calculations and simulations of simple evolutionary models that included directional selection, stabilizing selection, and mutational bias. Throughout these calculations, we suppose that although mutation rate can be biased towards trait-increasing or trait-decreasing alleles, the distribution of trait-increasing alleles is the same as that for trait-decreasing alleles.

Mutational bias alone

Mutational bias alone (*i.e.*, in the absence of selection) does not induce a dependency between $\bar{\beta}$ and DAF because mutations with differing βs are all equally likely to reach any given frequency. Hence, if the mutational bias is δ is the proportion of *de novo* mutations that are trait-increasing and the mean of the absolute value of a new mutation is $\mathbb{E}[|\beta|]$, then $\mathbb{E}[\beta] = \delta\mathbb{E}[|\beta|] - (1-\delta)\mathbb{E}[|\beta|]$. Note that when $\delta = 0.5$, mutation rates are symmetric, and $\mathbb{E}[\beta] = 0$.

Supposing that we decompose the frequency spectrum into k bins, when there is no selection $\mathbb{E}[S_\beta(0,1)] = \sum_{i=1}^{k} \mathbb{E}[\beta] = k\left(\delta\mathbb{E}[|\beta|] - (1-\delta)\mathbb{E}[|\beta|]\right)$. We perform this calculation for a variety of values of δ and a symmetric distribution of effect sizes, as well as stochastic simulations in which we select effect sizes randomly (Fig. S2), and observe excellent agreement between this simple calculation (solid lines) and the simulations (points).

$\mathbb{E}[S_\beta(0,x)]$ increases monotonically and linearly in x when mutational bias acts in the absence of selection on the trait, and the sign of S_β is always equal to the direction of the mutational bias (*i.e.*, mutational bias greater than 0.5 leads to positive S_β, while mutational bias less than 0.5 leads to negative S_β).

Polygenic adaptation

We next considered a toy model in which directional polygenic adaptation acts to increase the frequency of trait-increasing alleles. We suppose that trait-increasing alleles with effect size β have positive selection coefficients s with $\beta = s^\tau$, and that selection coefficients are drawn from a leptokurtic Γ-distribution peaked near 0. This model (which was proposed by Eyre-Walker for traits under negative selection (Eyre-Walker, 2010)) captures the idea that many alleles will have weak effects, and hence weak selective effects, while a small portion of alleles may have strong effects on the trait and be under relatively strong selection. We further suppose that the distribution and mutation rate of trait-increasing are the same as trait-decreasing alleles. We assume that the influence of binomial sampling is small and we do not account for it.

The time-dependent dynamics of the relationship between β and derived allele frequency are complex under such a model (because they depend on the transition probabilities from every possible initial frequency to every possible final frequency for all segregating selected alleles), but we can easily solve for the equilibrium state using diffusion theory. Under this model, the equilibrium frequency spectrum (i.e., the distribution of derived allele frequencies in the population at any given time) of trait increasing alleles is given by

$$f^+_{a,b}(x) = \int_\gamma \Gamma[a,b,\gamma] e^{-2\gamma} \frac{1-e^{2\gamma(1-x)}}{(e^{-2\gamma}-1)x(1-x)} d\gamma = \left(\frac{b}{4N}\right)^a \frac{\left(-\zeta[a,\frac{b}{4N}] + \zeta[a, 1-x+\frac{b}{4N}]\right)}{x(x-1)}, \tag{S1}$$

where $\Gamma[a,b,\gamma] = \frac{a^b}{\Gamma(a)}\gamma^{a-1}e^{-b\gamma}$, a and b are the parameters of the Γ-distribution, ζ is the Riemann zeta function, and γ is the population-scaled selection coefficient $2Ns$. Trait-decreasing alleles have a neutral frequency spectrum, which is given by $f^-_{a,b}(x) = \frac{1}{x}$. The mean value of β for trait increasing alleles at frequency x is given by

$$\beta^+_{a,b}(x) = \int_\gamma \frac{\Gamma[a,b,\gamma]\left(\frac{\gamma}{2N}\right)^\tau e^{-2\gamma}\frac{1-e^{2\gamma(1-x)}}{(e^{-2\gamma}-1)x(1-x)}}{f_{a,b}(x)} d\gamma = \frac{(4N)^{-\tau}\Gamma[a+\tau]\left(\zeta[a+\tau, 1+\frac{b}{4N}] - \zeta[a+\tau, x+\frac{b}{4N}]\right)}{\Gamma[a]\left(\zeta[a, 1+\frac{b}{4N}] - \zeta[a, x+\frac{b}{4N}]\right)}. \tag{S2}$$

For trait-decreasing derived alleles, the mean value of β does not depend on the derived allele frequency x (because we assume trait-decreasing alleles evolve neutrally) , and is given by

$$\beta^-_{a,b} = \int_\gamma \Gamma[a,b,\gamma]\left(\frac{\gamma}{2N}\right)^\tau d\gamma = (2Nb)^{-\tau}\frac{\Gamma[a+\tau]}{\Gamma[a]}. \tag{S3}$$

Combining terms and weighting by the frequency spectra for trait-increasing and trait-decreasing alleles, we find that the mean value of β as a function of DAF x ($\bar{\beta}(x)$) is given by

$$\beta_{a,b}(x) = \frac{(8b)^{-\tau} N^{-2\tau} \Gamma[a+\tau] \left(-(4N)^{a+\tau}(x-1) + b^{a+\tau}(-\zeta[a+\tau, \frac{b}{4N}] + \zeta[a+\tau, 1-x+\frac{b}{4N}])\right)}{\Gamma[a] \left(-(4N)^{a}(x-1) + b^{a}(-\zeta[a, \frac{b}{4N}] + \zeta[a, 1-x+\frac{b}{4N}])\right)}. \quad \text{(S4)}$$

We performed stochastic simulations under this model, in addition to our calculations. For the simulations, we used a weak distribution of selective effects ($\mathbb{E}[2Ns] \approx 8$) given by $\Gamma[a = 0.0415, b = 0.00515625]$, which was inferred from human conserved non-coding polymorphism data (Torgerson et al., 2009). Our results show that trait-increasing alleles will be more likely to increase in frequency (Fig. S1B), and will outnumber trait-decreasing alleles at all frequencies, resulting in $\beta_{a,b}(x) > 0$ at all frequencies (Fig. S1C). This implies that under this simple polygenic adaptation model, the S_β will increase monotonically in the direction of the selection (*i.e.*, if selection favors increases in trait values, $S_\beta(0, x)$ will be positive and monotonically increasing in x, while $S_\beta(0, x)$ will be negative and monotonically decreasing in x if lower trait values are preferred). Our analytical calculations from eqn. S4 (Fig. S1C, black curve) are in tight agreement with the simulations (Fig. S1C, points). Equations in this section were solved using *Mathematica*.

Mutational bias and negative selection

Negative selection against trait-altering alleles will also generate an increasing or decreasing βDAF relationship, if trait-increasing and trait-decreasing mutations are not equally likely to occur. To better understand this relationship quantitatively, we used analytical calculations under Eyre-Walker's quantitative trait model to calculate the relationship between β and DAF.

We define mutational bias δ as the proportion of new mutations that are trait-increasing, and as in the previous section, we suppose that effect sizes are given by $\beta = |s|^\tau$. In contrast to the previous section (but in accordance with the original Eyre-Walker model), we suppose that all causal alleles are deleterious, regardless of their direction of effect. We suppose that s is drawn from a Γ-distribution.

Under this model, we are able to calculate $\beta_{a,b}(x)$ using the same procedure as in the previous section. We find

$$\beta_{a,b}(x) = (1-2\delta) \frac{4N^{-\tau} \Gamma[a+\tau] \left(-\zeta[a+\tau, 1+\frac{b}{4N}] + \zeta[a+\tau, x+\frac{b}{4N}]\right)}{\Gamma[a] \left(-\zeta[a, 1+\frac{b}{4N}] + \zeta[a, x+\frac{b}{4N}]\right)}. \quad \text{(S5)}$$

We performed stochastic simulations under this model for a variety of values of δ, again using a weak distribution of selection coefficients that was fit to human conserved non-coding sequences and $\tau = 0.5$ (Torgerson et al., 2009). We find excellent agreement between our simulations and the analytical predictions (Fig. S3). When $\delta = 0.5$, trait-increasing and trait-decreasing alleles are exactly balanced, and there is no dependency of β on DAF. In contrast, as δ becomes increasingly biased, trait-decreasing alleles further outnumber trait-increasing alleles (Fig. S3). Since larger effect alleles are subject to stronger selection, the difference is greater at low frequency than high frequency, generating a correlation between β and DAF. Taking $S_\beta(0, x)$ as the sum over the terms in eqn. S5, we find that S_β increases monotonically in magnitude in the same direction as the mutation bias under this model. In contrast to the mutational-bias only model, $S_\beta(0, x)$ does not increase linearly in magnitude as a function of x when selection acts.

Stabilizing selection with shifts

The previous sections show that a βDAF plot and S_β can capture signals of positive and negative selection as well mutational bias, but there is now substantial evidence that both positive and negative selection may act on complex traits. Indeed, it seems unlikely that polygenic adaptation will continue indefinitely over long evolutionary times, because this would imply that the trait would continue to increase or decrease over very long timescales. A more natural way to model negative selection punctuated with periods of adaptation is through stabilizing selection with shifts in the fittest value of the phenotype (Jain and Stephan, 2017).

We develop a polygenic selection quantitative trait model that maps selection coefficients s to effect sizes β using the well-established Gaussian stabilizing selection model (Robertson, 1956; Barton, 1986). We suppose

that stabilizing selection acts on a trait, and that the fittest value of the trait is ϕ_o (also referred to as the "trait optimum"), such that the fitness f of an individual with trait value ϕ is given by

$$f(\phi) = e^{\frac{-(\phi-\phi_o)^2}{w^2}}, \tag{S6}$$

where w is the breadth of the fitness function. We additionally suppose that the trait ϕ has a normal distribution such that

$$P(\phi) = \frac{1}{\sqrt{2\pi\sigma^2}} e^{\frac{-(\phi-\bar{\phi})^2}{\sigma^2}}, \tag{S7}$$

where σ is the standard deviation of the fitness distribution and $\bar{\phi}$ is the mean trait value in the population. Under these conditions, it is possible to solve for the per-generation, per-individual selection coefficient s as a function of the above model parameters for causal alleles of effect size β. We calculate s by marginalizing the fitness effect of a new mutation of effect size β across all fitness backgrounds. Our calculation proceeds similarly to previous work (Barton, 1986; Simons et al., 2018), although we retain the dependence on the current mean phenotype value ($\bar{\phi}$) and the phenotype optimum (ϕ_o), rather than assuming that the phenotype distribution is centered at the optimum. While the full expression for the expected change in allele frequency is provided in the next section, we note that when the trait is at equilibrium such that $\bar{\phi} = \phi_o$,

$$s \approx -\frac{\beta^2}{2\left(\sigma^2 + w^2\right)}, \tag{S8}$$

and the expected frequency change δp for an allele at frequency p with effect size β when $\bar{\phi} = \phi_o$ is

$$\mathbb{E}[\delta p] \approx -\frac{\beta^2}{2\left(\sigma^2 + w^2\right)} p(1-p)(-1+2p), \tag{S9}$$

which implies that the trait evolves as if underdominant when the mean population trait value is centered close to the optimum.

Recasting the selection coefficient in terms of the trait distribution

To calculate the time-dependent selection coefficient $s(t)$ of a site with effect size β, we first develop some results that allow us to recast the selection coefficient s at equilibrium as a function of the proportion of the population in which an allele of effect size β is fitness-increasing. Since the trait is normally distributed, the probability that an individual has phenotype value ϕ, $P(\phi)$, is given by $P(\phi) = \frac{1}{\sqrt{2\pi\sigma^2}} e^{\frac{-(\phi-\bar{\phi})^2}{\sigma^2}}$. To calculate the selection coefficient for an allele with effect size β, we then marginalize across all trait backgrounds and possible genotypes in the population to obtain the mean fitness effect, since the fitness effect varies across trait backgrounds according to $f(\phi)$. The three possible genotypes for a causal variable allele are denoted as aa (homozygous ancestral), ad (heterozygous), and dd (homozygous derived). For each genotype, we calculate the fitness w of an allele at frequency p as

$$w_{aa} = \int_{-\infty}^{\infty} f(\phi) P(\phi + 2p\beta) d\phi, \tag{S10}$$

$$w_{ad} = \int_{-\infty}^{\infty} f(\phi + \beta) P(\phi + 2p\beta) d\phi, \tag{S11}$$

$$w_{dd} = \int_{-\infty}^{\infty} f(\phi + 2\beta) P(\phi + 2p\beta) d\phi. \tag{S12}$$

substituting in $P(\phi)$ and $f(\phi)$, these integrals can be solved to obtain $w_{aa} \propto e^{\frac{-(2p\beta+\phi_o-\bar{\phi})^2}{2(w^2+\sigma^2)}}$, $w_{ad} \propto e^{\frac{-((-1+2p)\beta+\phi_o-\bar{\phi})^2}{2(w^2+\sigma^2)}}$, and $w_{dd} \propto e^{\frac{-(2(-1+p)\beta+\phi_o-\bar{\phi})^2}{2(w^2+\sigma^2)}}$. We then can solve for the expected change in frequency $\mathbb{E}[\delta p]$ at time of an allele at frequency p as

$$\mathbb{E}[\delta p] = \frac{p(1-p)w_{ad} + p^2 w_{dd}}{2p(1-p)w_{ad} + p^2 w_{dd} + (1-p)^2 w_{aa}} - p, \tag{S13}$$

Taking a series expansion about $\beta = 0$, we find that

$$\mathbb{E}[\delta p] \approx \frac{\beta\left(\phi_o - \bar{\phi}\right) p\,(1-p)}{w^2+\sigma^2} - \frac{\beta^2\left(p(1-p)(2p-1)\left(w^2+\sigma^2-\left(\bar{\phi}-\phi_o\right)^2\right)\right)}{2(w^2+\sigma^2)^2} \tag{S14}$$

When $\bar{\phi} = \phi_o$, alleles evolve as if underdominant because $\mathbb{E}[\delta p] \approx -\frac{\beta^2}{2\left(\sigma^2+w^2\right)}p(1-p)(-1+2p)$. When $\bar{\phi} \neq \phi_o$, alleles may either be expected to increase in fitness (*i.e.*, be transiently positively selected) or decrease in frequency, depending on their effect size and the current mean population trait value $\bar{\phi}$. In our Wright-Fisher simulations, we replace the expected frequency change with eqn. S14 and track the current mean population trait value as a time-dependent quantity (see next section for more details).

Validation of simulations

We developed a custom simulator of our model in Python. Our simulator accommodates changes in population size, including explosive growth and bottlenecks, as well as any arbitrary distribution of selection coefficients. We perform a burn-in period of $5N$ generations for a simulated population of size N, during which we suppose that the population is close to equilibrium and hence s does not vary each generation. After the first burn-in, we perform an additional $5N$ generations of burn-in during which we recalculate $\mathbb{E}[\delta p]$ as a function of β, $\bar{\phi}$, and ϕ_o in each generation with eqn. S14. To calculate the current value of $\bar{\phi}$, we sum over all j causal alleles, such that $\bar{\phi} = \sum_j 2q_j\beta_j$, where q_j is the frequency of a site with effect size β_j.

For simulations of polygenic adaptation, at some time t_s during the simulations, we reset ϕ_o to a new value, which induces the trait distribution to be out-of-equilibrium with respect to the fitness function. During the out-of-equilibrium period, a portion of the causal sites will be positively selected (specifically those that are fitness increasing when marginalizing across all trait backgrounds, as described in the previous section), while the remaining sites will be fitness-decreasing. Each generation, we recalculate δp based on the current configuration of $\bar{\phi}$ and ϕ_o.

To validate our simulator, we simulated a complex selection and demographic model using previously published models of European demographic history (Gravel et al., 2011) and selection on human conserved elements (Torgerson et al., 2009) in SFS_CODE (Hernandez, 2008) and compared the SFS_CODE frequency spectrum to the frequency spectrum in our simulations. When no shift in the trait optimum occurs, selection coefficients in our model have the same expected value as in the standard Wright-Fisher model with underdominance, so the frequency spectra should be similar. However, since underdominance is not straightforward to simulate in SFS_CODE, for this set of validation simulations we replaced the underdominance term in our simulations with the standard genic selection model. Results of these simulations are plotted in Fig. S4. We observe good agreement between the two spectra overall, although our model results in a slight over-representation of rare alleles and a slight under-representation of common alleles relative to SFS_CODE. Note that there is weak LD in the SFS_CODE simulations and that our selection model differs slightly, so we do not expect perfect agreement. For the SFS_CODE simulations, we used the following command line: `sfs_code 1 500 -N 1000 -n 100 -A -t 0.001 -r 0.0 -TE 0.405479 -Td 0 P 0 1.982738 -Td 0.265753 P 0 0.128575 -Td 0.342466 P 0 0.554541 -Tg 0.342466 P 0 55.48 -L 100 150 -l g 0.5 R -a N R -W 2 0 1 1 0.0415 0.00515625 -s <random_seed> -o <out>`

For the simulations presented in Fig. 1, the curves represent the mean over 100 independent simulations. We used $N = 7,000$ (where N is the ancestral population size) for the simulations in Fig. 1., but rescaled the population size for computational efficiency to 2,000 for parameter inference (see next section). The simulation code was implemented in Python and numpy, and will be freely available and posted on Github.

S_β in simulation

We performed simulations of Gaussian stabilizing selection with shifts in the optimal phenotype value for a wide range of parameter combinations. Since we ultimately apply our results to data from European GWAS, we apply a European demographic model (including ancestral expansion and bottleneck events, as well as recent exponential growth) that was fit to patterns of genomic diversity (Gravel et al., 2011). Our goal in these simulations is to understand the qualitative behavior of S_β as a function of evolutionary parameters (Fig. 1C-E), and in subsequent sections we develop a more quantitative approach for mapping evolutionary parameters to observed patterns in GWAS summary statistics.

For simulations shown in Fig. 1, we used $N = 7,000$ (in accordance with (Gravel et al., 2011)), a mutational bias of $\delta = 0.4$, $h^2 = 0.8$, and selection strength $\omega = 1$. Effect sizes drawn from a Gamma distribution with $a = 0.037$ and $b = 0.0008$, and assumed a genome-wide polygenicity of 0.02 (*i.e.*, 2% of target sites have a non-zero effect on phenotype). While these choices are arbitrary, we use rejection sampling to find parameter combinations that fit the observed data in the subsequent section.

On the advice of a reviewer, we also explored more gradual and less dramatic shifts than those we simulated in Fig. 1. We simulated shifts of 0.5, 1, and 2 trait standard deviations that occur linearly over 100 generations (*e.g.*, the optimum increases by $^{0.5}/_{100}$ per generation in the 0.5 case), but otherwise with the same parameters as above. We performed this set of experiments to confirm that the dynamics we observe are not driven primarily by unrealistically abrupt shifts that would not be observed in nature. We found that a gradual shift with $\Delta\phi = 2$ had nearly identical patterns to the instantaneous case presented in Fig. 1 (Fig. S19A-C). However, reducing the total magnitude to 1 and 0.5 trait standard deviations drives substantial decreases in the magnitude of the signal (Fig. S19D-I). As expected, these results imply that smaller and more ancient shifts will be more difficult to detect, but instantaneous shifts drive similar patterns to gradual shifts (at least in this part of the parameter space).

Inferring evolutionary parameters

We developed a rejection-sampling based approach to infer evolutionary parameters (Tavaré et al., 1997). Rejection sampling uses informative summary statistics in model-based simulations to infer an approximate posterior distribution of model parameters. Simulations are performed under the model with parameters drawn from a wide range of possible combinations, and then the subset of simulations that most closely match the observed data are retained to build the posterior. The remaining simulations are rejected.

We performed simulations of Gaussian stabilizing selection with shifts in the optimal phenotype value using parameter values that were sampled from broad prior distributions, and we fit these simulated data to the observed $S_\beta(0.01, x)$ data for our phenotypes (we exclude alleles below 1% frequency to avoid the potential effects of rare variant stratification). As summary statistics, we take $S_\beta(0.01, x)$ for $x \in \{0.02, 0.03, 0.04, 0.07, 0.12, 0.22, 0.52, 0.62, 0.72, 0.82, 0.92\}$. Additionally, since the absolute magnitude of S_β depends on the units of the effect sizes, we defined scaled $S_\beta(x_i, x_f)$ as $\frac{S_\beta(x_i,x_f)}{S_\beta(0.01,0.99)}$, and use the scaled summary statistics as input to our method. As a final summary statistic, we take $\frac{S_\beta(0.01,0.99)}{k\bar{\beta}}$, where $\bar{\beta}$ is the mean effect size over all effect sizes in the study (or simulation) and k is the number of frequency bins ($k = 100$ here). As we showed earlier in the Supplemental Information, S_β has expectation $k\bar{\beta}$ when only mutational bias acts on the trait, so this summary statistic has expectation 1 when only mutational bias acts on the trait.

We performed 10^5 simulations, drawing evolutionary parameters from very wide prior distributions. Parameters of the model included heritability (h^2, uniform from 0.5 to 1), shift in optimum phenotype ($\Delta\phi$, uniform on -5 to 5 in units of the standard deviation of the ancestral trait distribution), polygenicity (Ψ, chosen uniformly from 0.004 to 0.02 of sites genome-wide being causal), effect size distribution parameters (chosen from the square root of a Γ-distributed according to a and b, with a chosen to be within a factor of 4 greater or less than 0.04 and b chosen to be within a factor of 4 of 8e-5 – this enforces selection coefficients to be drawn from a Γ-distribution), mutational bias δ (uniform on 0.25 to 0.75), the time of the shift in optimal phenotype (t_s, uniform from -1 to 0.4 in coalescent units, corresponding to the time period ranging from 500,000 to 17,500 years ago), and ancestral uncertainty (unifom from 0.7 to 1, where 1 represents perfect ancestral assignment). Because we vary both heritability and the effect size distribution, we did not additionally vary the selection parameter of Gaussian stabilizing selection ω, which we set at 1. We rescaled the population size to $N = 2 \times 10^3$ for computational efficiency (the ancestral population size in the demographic model is $N = 7 \times 10^3$). Although we do not directly vary the selection coefficient distribution (because selection coefficients depend on effect sizes and the current trait variance σ^2, and we sample effect size parameters as opposed to selection parameters), our simulations include both very weak selection per site and strong selection per site. After each simulation finishes, we mix the causal alleles with neutral alleles that are simulated under the same demographic model, and have effect size 0, representing the portion of the genome that is not causal for the trait but is included in the GWAS. We calculate the sum of squared differences between our simulated and observed statistics for all 10^5 simulations, and retain the 0.25% of simulations that minimize this statistic.

To test our rejection sampling method, we masked the input parameter set of each simulation and attempted

to infer the parameter values that were used to run the simulation. We used the maximum *a posteriori* estimate as a point estimate of the parameter value for these experiments. We found high predictability of the direction of both mutational bias δ and $\Delta\phi$ (Fig. 3C&D), and noisy estimation of the magnitude of these parameters. Most other parameters were poorly predicted. We found that the true timing of selection events was highly correlated with the inferred timing, but a large number of outlier estimates make the estimates of this parameter less trustworthy, so we do not report it. All other parameters were inferred with low accuracy, hence we only report δ and $\Delta\phi$.

In addition to the power to detect mutation rate bias (δ) and shifts in optimal phenotype value ($\Delta\phi$), we also considered the false positive rate for detection of these parameters (Fig. S5). We examined the subset of simulations among our 10^5 that had $|\Delta\phi| < 0.25$ and the subset with $0.45 < \delta < 0.55$ and assessed the proportion of these simulations for which we estimated maximum *a posteriori* parameters larger than y for all estimated values y. We found that the false positive rate is high for both small mutation rate biases and weak shifts, but low for effects as large as those we infer in the data, implying that our method is unlikely to result in a multiple false positives for shifts and mutation rate biases. Note that this null is very conservative, because all of the simulations we used to assess false positive rate include stabilizing selection, and the δ false positive simulations include shifts in selection while the $\Delta\phi$ false positive simulations include bias in mutation rate.

It should be noted that while our empirical selection detection method incorporates linkage, our rejection sampling method does not. Linkage between causal alleles and non-causals could cause the data to have larger deviations from 0 in S_β than our simulations, possibly causing our estimates of the parameters to be upwardly biased. For this reason (and because the estimates of parameters are noisy in our simulations), we focus primarily on the direction of the effects rather than their magnitudes.

Empirical data analyses

Aggregating phenotype data

We obtained GWAS summary data from several published studies for nine different phenotypes as discussed in the main text (Wood et al., 2014; Shungin et al., 2015; Locke et al., 2015; CDG Psychiatric Genomics Consortium, 2013; Franke et al., 2010; Day et al., 2015; Global Lipids Genetics Consortium et al., 2013; Okbay et al., 2016). We obtained allele frequencies for each allele in each study from the GWAS summary data in the corresponding study. To calculate S_β for each study, we first needed to polarize all the alleles by their derived/ancestral status. We obtained inferred derived/ancestral states for each allele from the 1000 Genomes project (Thousand Genomes Project Consortium et al., 2015). Our permutation method requires that each allele then be assigned to a LD block within the genome (Berisa and Pickrell, 2016). To assign each of these alleles to LD blocks, we also used the 1000 Genomes data to obtain the genomic coordinates for each allele. Alleles for which we could not assign states or LD blocks were excluded.

Calculating S_β and implementing our empirical framework

To account for LD in selection tests for complex traits using GWAS summary data, we divided the genome into 1,703 LD blocks, which were previously identified as being approximately independent (Berisa and Pickrell, 2016). For each LD block, we then select a random sign (positive or negative with equal probability), and multiply all the effect sizes in the LD block by this sign. We then recompute summary statistics (such as the correlation between frequency and MAF) on the randomized data. By repeating this procedure, we generate a null distribution for the test statistic. This method maintains the correlations between effect sizes generated by LD, the site frequency spectrum of the sampled alleles, and the joint distribution of the absolute value of effect size and allele frequency, while breaking any relationship between effect sizes and allele frequency. Note that this is a conservative permutation, because many of the alleles within an LD block are not linked or only weakly linked. We further consider the robustness of our method to population stratification in a subsequent section, which is a persistent potential source of false positives for studies of selection. To assess significance, we perform a two-tailed test comparing the observed value of the test statistic to the permutation-based null distribution. We performed 2,000 permutations for each phenotype.

We developed custom software implementing our approach in Python, which is freely available upon request and will be posted on Github. To calculate S_β, we group alleles into 1% frequency bins – without performing this

grouping, many high frequency bins would have very few alleles and very noisy estimates of $\overline{\beta}$. We selected 1% because it strikes a balance between obtaining low standard errors on $\overline{\beta}$ and finely parsing the allele frequency space, but we note that other choices of bin size could potentially improve our power.

Replication data & analysis

As a replication cohort for our top three selection signals, we obtained GWAS summary statistics from the UK Biobank (UKBB) from https://data.broadinstitute.org/alkesgroup/UKBB/ (height, BMI, and educational attainment – note that approximately 1/4 of the UKBB samples were included in Okbay *et al*, so the studies are not completely independent). We used our framework to calculate S_β on each phenotype, and estimate the linkage-adjusted variance in the test-statistic. We perform a one-tailed test for significance, since we have an expectation for the sign of S_β from our first set of findings. We replicate our findings for both BMI ($p = 0.0095$) and educational attainment ($p < 5e-4$) (Fig. S18). Perhaps surprisingly, height did not replicate (see main text for discussion of possible explanations).

In addition to S_β, we performed a replication study at signals of selection that were used in previous projects to identify signals of selection on height in the GIANT study. One study identified a positive correlation between effect size and allele frequency (Yang et al., 2015), while another found a correlation of allele frequency difference between northern and southern Europe and the p-value rank of the height increasing-allele (Turchin et al., 2012). Neither of these correlations were replicated in the UK Biobank summary statistics (Fig. S6).

Neanderthal alleles

A list of Neanderthal alleles that were inferred with the S^* statistic (Plagnol and Wall, 2006) was generously provided by Rajiv McCoy and Josh Akey. In analyses using Neanderthal alleles, we masked all alleles that were not present in this dataset. The dataset includes 139,694 SNPs, 139,381 of which we were able to map to rs numbers and use in our analysis.

Neanderthal polarization and alternate summary statistics. Although we have highlighted the ancestral/derived polarization, other polarizations, such as Neanderthal vs ancestral human alleles, can also provide insight into evolutionary processes. Recent evidence suggests that Neanderthal immune-related variants and expression-altering variants were likely targets of selection (Quach et al., 2016; Nédélec et al., 2016; McCoy et al., 2017), and some Neanderthal variants are likely to alter complex traits (Simonti et al., 2016), but little is known about polygenic selection on trait-altering Neanderthal alleles. If Neanderthals were "pre-adapted" to Europe, alleles that were fixed in Neanderthals may have accelerated the adaptation of modern humans to the European environment when admixed into humans (Enard and Petrov, 2018). Hence, we hypothesized that we may observe signals in S_β if alleles that fixed on the Neanderthal lineage were systematically biased in one direction than the other, or if selection acted to promote or remove Neanderthal alleles with specific phenotypic effects once admixed into modern humans.

We tested this hypothesis by computing S_β on Neanderthal alleles that were included in previous GWAS. The alleles were identified with the S^* statistic (Plagnol and Wall, 2006) and allele sharing with Neanderthal genomes, and hence do not suffer from the same potential biases from ancestral uncertainty as the derived/ancestral polarization. We performed both a common allele test (S_β(0.05,0.25)) and an all allele test (S_β(0,0.25)) for each of the nine phenotypes. We exclude alleles above frequency 0.25 because the vast majority of Neanderthal alleles have frequencies below 0.25.

Across the nine phenotypes we studied, we found signals for both height (p <5e-4) and major depression (p <5e-4) (Fig. S18), and a marginally significant signal for schizophrenia that did not pass a multiple testing correction (p =0.015). The height and schizophrenia signals were strongest when including only common alleles, whereas the depression signal was driven primarily by rare alleles. The height signal suggests selection that favored Neanderthal height-increasing alleles (either on the Neanderthal lineage or in modern humans), while the depression signal suggests that Neanderthal alleles had a large impact on depression risk, which have been preferentially pushed to low frequency by selection. The schizophrenia signal suggests selection against schizophrenia risk or a bias towards protective alleles from Neanderthals. No other phenotypes had significant p-values.

The impact of ancestral uncertainty on S_β

Ancestral states are not directly observed in genomic data, and are typically inferred by comparing human sequences to those of an out-group. The underlying assumption is that the allelic state in the out-group represents the most likely ancestral state for the allele. While this approach correctly assigns the ancestral state for the majority of alleles, it does not account for recurrent fixation events at a single site, leading to some rate of ancestral misassignment. Since our method to detect selection compares ancestral and derived alleles, we sought to understand how ancestral misassignment would impact our inferences.

Ancestral misassignment will tend to decrease the absolute value of S_β within each frequency bin, and hence decrease our power. To see this, suppose that for a given minor allele frequency x, we misassign ancestral state with probability $1-p$, and $\Psi(x)$ is the number of derived alleles observed at frequency x. We note that we can rewrite S_β as the difference in mean beta between ancestral and derived alleles of equal minor allele frequency (*i.e.*, $S_\beta = \sum_x \bar{\beta}_D(x) = \sum_x \frac{1}{2}\left(\bar{\beta}_D(x) - \bar{\beta}_A(x)\right)$. The expected value of the the test statistic within this bin is then

$$\mathbb{E}[S_\beta(x)] = \frac{1}{2}\left(\left(p\Psi(x)\overline{\beta}_D + (1-p)\Psi(1-x)\overline{\beta}_A\right) - \left(p\Psi(1-x)\overline{\beta}_A + (1-p)\Psi(x)\overline{\beta}_D\right)\right), \quad \text{(S15)}$$

where β_D is the effect size of derived alleles and β_A is the effect size of ancestral alleles. Note that if $p = 0.5$, we are randomly guessing at ancestral states, and $\mathbb{E}[S_\beta(x)] = 0$. If $p < 0.5$, then the absolute value of $\mathbb{E}[S_\beta(x)]$ is strictly less than it's true value (*i.e.*, the value that would be observed with no ancestral uncertainty).

While this analysis shows that ancestral uncertainty serves to make our analyses more conservative, we also sought to understand its potential impact on the observed relationship between allele frequency and effect size (Fig. S8). Because $\Psi(x)$ is generally much larger than $\Psi(1-x)$ for $x < 0.5$ (*i.e.*, there are more rare alleles than common alleles in genomic sequencing data), we expect that the impact of ancestral uncertainty will be greatest at very high derived allele frequencies (*e.g.*, $x > 0.9$). We fit a linear model by regressing the mean effect size on allele frequency for the observed height data for derived alleles in the frequency range 40-60%, and extrapolated this curve out to 100% frequency, supposing that frequencies near 50% were only modestly impacted by ancestral uncertainty. We then simulated the impact of ancestral uncertainty at various levels from $p = 1\%$ to $p = 10\%$. At $p = 10\%$ uncertainty we see a striking resemblance between our simulated data and the observed data. We conclude that moderate levels of ancestral uncertainty are likely responsible for the "*S*" shaped curve that we observe for many of our phenotypes.

References

NH Barton. The maintenance of polygenic variation through a balance between mutation and stabilizing selection. *Genetics Research*, 47(3):209–216, 1986.

Tomaz Berisa and Joseph K Pickrell. Approximately independent linkage disequilibrium blocks in human populations. *Bioinformatics*, 32(2):283–285, 2016.

Felix R Day, Katherine S Ruth, Deborah J Thompson, Kathryn L Lunetta, Natalia Pervjakova, Daniel I Chasman, Lisette Stolk, Hilary K Finucane, Patrick Sulem, Brendan Bulik-Sullivan, et al. Large-scale genomic analyses link reproductive aging to hypothalamic signaling, breast cancer susceptibility and BRCA1-mediated DNA repair. *Nature Genetics*, 47(11):1294–1303, 2015.

David Enard and Dmitri A Petrov. Evidence that rna viruses drove adaptive introgression between neanderthals and modern humans. *Cell*, 175(2):360–371, 2018.

Adam Eyre-Walker. Genetic architecture of a complex trait and its implications for fitness and genome-wide association studies. *Proceedings of the National Academy of Sciences*, 107(suppl 1):1752–1756, 2010.

Andre Franke, Dermot PB McGovern, Jeffrey C Barrett, Kai Wang, Graham L Radford-Smith, Tariq Ahmad, Charlie W Lees, Tobias Balschun, James Lee, Rebecca Roberts, et al. Genome-wide meta-analysis increases to 71 the number of confirmed crohn's disease susceptibility loci. *Nature Genetics*, 42(12):1118–1125, 2010.

Simon Gravel, Brenna M Henn, Ryan N Gutenkunst, Amit R Indap, Gabor T Marth, Andrew G Clark, Fuli Yu, Richard A Gibbs, Carlos D Bustamante, David L Altshuler, et al. Demographic history and rare allele sharing among human populations. *Proceedings of the National Academy of Sciences*, 108(29):11983–11988, 2011.

Ryan D Hernandez. A flexible forward simulator for populations subject to selection and demography. *Bioinformatics*, 24(23):2786–2787, 2008.

Kavita Jain and Wolfgang Stephan. Rapid adaptation of a polygenic trait after a sudden environmental shift. *Genetics*, 206(1):389–406, 2017.

Adam E Locke, Bratati Kahali, Sonja I Berndt, Anne E Justice, Tune H Pers, Felix R Day, Corey Powell, Sailaja Vedantam, Martin L Buchkovich, Jian Yang, et al. Genetic studies of body mass index yield new insights for obesity biology. *Nature*, 518(7538):197–206, 2015.

Rajiv C McCoy, Jon Wakefield, and Joshua M Akey. Impacts of neanderthal-introgressed sequences on the landscape of human gene expression. *Cell*, 168(5):916–927, 2017.

Yohann Nédélec, Joaquín Sanz, Golshid Baharian, Zachary A Szpiech, Alain Pacis, Anne Dumaine, Jean-Christophe Grenier, Andrew Freiman, Aaron J Sams, Steven Hebert, et al. Genetic ancestry and natural selection drive population differences in immune responses to pathogens. *Cell*, 167(3):657–669, 2016.

Aysu Okbay, Jonathan P Beauchamp, Mark Alan Fontana, James J Lee, Tune H Pers, Cornelius A Rietveld, Patrick Turley, Guo-Bo Chen, Valur Emilsson, S Fleur W Meddens, et al. Genome-wide association study identifies 74 loci associated with educational attainment. *Nature*, 533(7604):539–542, 2016.

Vincent Plagnol and Jeffrey D Wall. Possible ancestral structure in human populations. *PLoS Genetics*, 2(7): e105, 2006.

Hélène Quach, Maxime Rotival, Julien Pothlichet, Yong-Hwee Eddie Loh, Michael Dannemann, Nora Zidane, Guillaume Laval, Etienne Patin, Christine Harmant, Marie Lopez, et al. Genetic adaptation and neandertal admixture shaped the immune system of human populations. *Cell*, 167(3):643–656, 2016.

Alan Robertson. The effect of selection against extreme deviants based on deviation or on homozygosis. *Journal of Genetics*, 54(2):236–248, 1956.

Dmitry Shungin, Thomas W Winkler, Damien C Croteau-Chonka, Teresa Ferreira, Adam E Locke, Reedik Mägi, Rona J Strawbridge, Tune H Pers, Krista Fischer, Anne E Justice, et al. New genetic loci link adipose and insulin biology to body fat distribution. *Nature*, 518(7538):187–196, 2015.

Yuval B Simons, Kevin Bullaughey, Richard R Hudson, and Guy Sella. A population genetic interpretation of gwas findings for human quantitative traits. *PLoS Biology*, 16(3):e2002985, 2018.

Corinne N Simonti, Benjamin Vernot, Lisa Bastarache, Erwin Bottinger, David S Carrell, Rex L Chisholm, David R Crosslin, Scott J Hebbring, Gail P Jarvik, Iftikhar J Kullo, et al. The phenotypic legacy of admixture between modern humans and neandertals. *Science*, 351(6274):737–741, 2016.

Simon Tavaré, David J Balding, Robert C Griffiths, and Peter Donnelly. Inferring coalescence times from dna sequence data. *Genetics*, 145(2):505–518, 1997.

CDG Psychiatric Genomics Consortium. Identification of risk loci with shared effects on five major psychiatric disorders: a genome-wide analysis. *The Lancet*, 381(9875):1371–1379, 2013.

Global Lipids Genetics Consortium et al. Discovery and refinement of loci associated with lipid levels. *Nature Genetics*, 45(11):1274–1283, 2013.

Thousand Genomes Project Consortium et al. A global reference for human genetic variation. *Nature*, 526(7571): 68–74, 2015.

Dara G Torgerson, Adam R Boyko, Ryan D Hernandez, Amit Indap, Xiaolan Hu, Thomas J White, John J Sninsky, Michele Cargill, Mark D Adams, Carlos D Bustamante, et al. Evolutionary processes acting on candidate cis-regulatory regions in humans inferred from patterns of polymorphism and divergence. *PLoS Genetics*, 5(8):e1000592, 2009.

Michael C Turchin, Charleston WK Chiang, Cameron D Palmer, Sriram Sankararaman, David Reich, Joel N Hirschhorn, Genetic Investigation of ANthropometric Traits (GIANT) Consortium, et al. Evidence of widespread selection on standing variation in europe at height-associated snps. *Nature Genetics*, 44(9):1015–1019, 2012.

Lawrence H Uricchio, Noah A Zaitlen, Chun Jimmie Ye, John S Witte, and Ryan D Hernandez. Selection and explosive growth alter genetic architecture and hamper the detection of causal rare variants. *Genome Research*, 26(7):863–873, 2016.

Andrew R Wood, Tonu Esko, Jian Yang, Sailaja Vedantam, Tune H Pers, Stefan Gustafsson, Audrey Y Chu, Karol Estrada, Jian'an Luan, Zoltán Kutalik, et al. Defining the role of common variation in the genomic and biological architecture of adult human height. *Nature Genetics*, 46(11):1173–1186, 2014.

Jian Yang, Andrew Bakshi, Zhihong Zhu, Gibran Hemani, Anna AE Vinkhuyzen, Sang Hong Lee, Matthew R Robinson, John RB Perry, Ilja M Nolte, Jana V van Vliet-Ostaptchouk, et al. Genetic variance estimation with imputed variants finds negligible missing heritability for human height and body mass index. *Nature Genetics*, 47:1114–1120, 2015.

Supplemental Figures

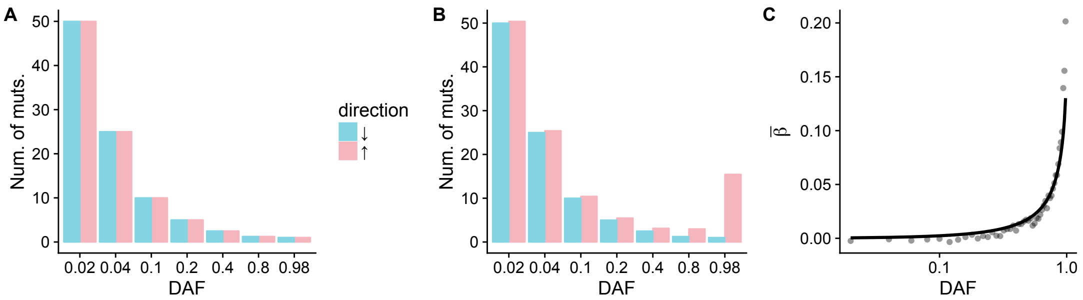

Figure S1: A: When selection does not prefer either trait-increasing or trait-decreasing alleles, and mutation bias does not act on a trait, then trait-increasing (pink) and -decreasing (blue) alleles are expected to have identical frequency spectra and equal mean β values within each frequency bin. B: When selection acts to increase a trait, trait-increasing alleles will increase in frequency and persist longer than trait-decreasing alleles, resulting in a relationship between $\bar{\beta}$ and frequency. C: $\bar{\beta}$ vs DAF for a toy model of where trait-increasing alleles are favored. The black line represents an analytical calculation (eqn. S4) while the black points are the results of stochastic simulations.

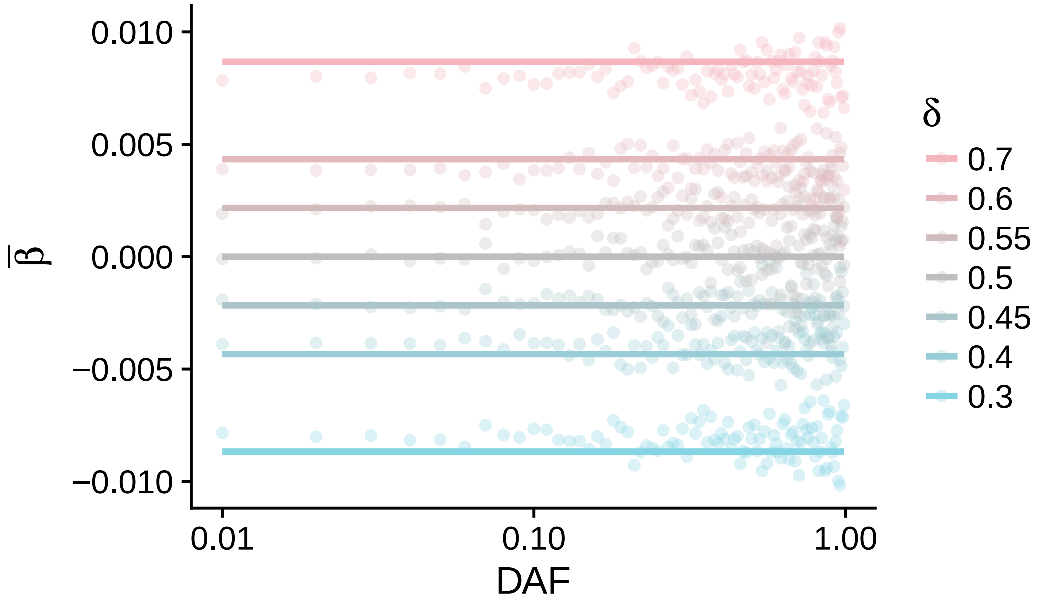

Figure S2: The relationship between β and DAF when only mutational bias acts on the trait (*i.e.*, there is no selection). Mean β is shifted in the direction of the bias, but does not depend on DAF.

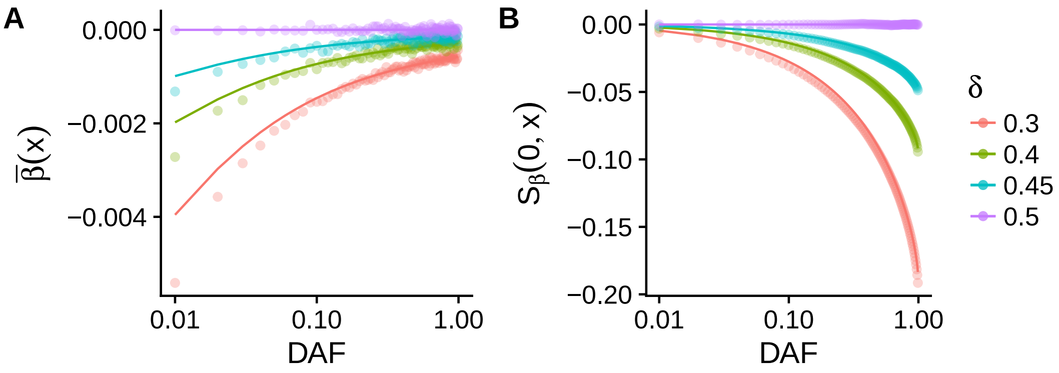

Figure S3: A: A βDAF plot for various values of mutational bias (δ) towards trait decreasing alleles for traits under selection. B: $S_\beta(0, x)$ for the same models in A. The solid lines represent the results of analytical calculations (eqn. S5) while the points represent stochastic simulations.

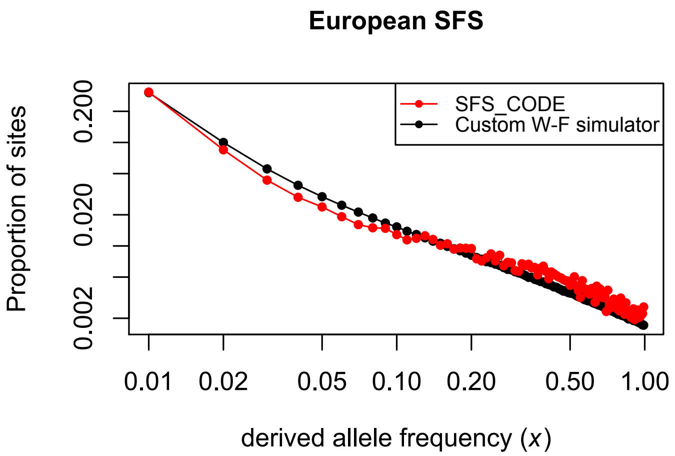

Figure S4: A comparison of simulated frequency spectra for a complex demographic/selection model for our Wright-Fisher simulator (black) and SFS_CODE (red).

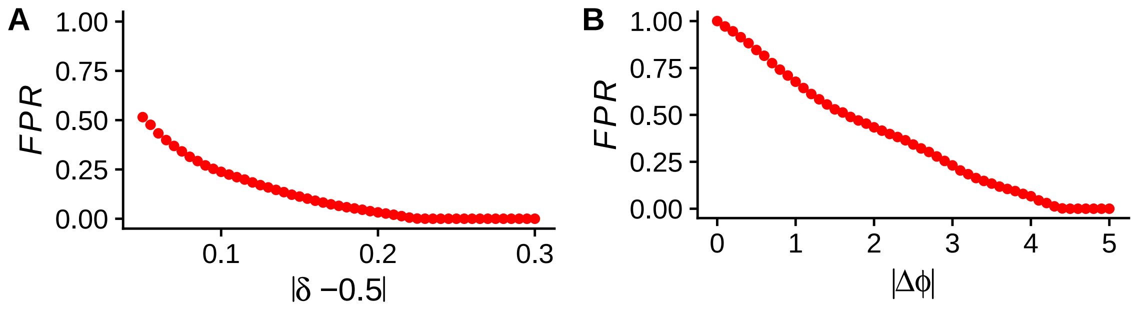

Figure S5: Conservative False Positive Rate estimation for detection of mutation rate bias and shifts in optimal phenotype value. We plot the estimated probability (FPR) that the inferred parameter exceeds the value on the x-axis, given that no mutation bias (A) or shift in optimal phenotype (B) occurred. As expected, it is not unlikely to infer a small shift in cases when no shift occurred, but it is highly unlikely to infer a large shift.

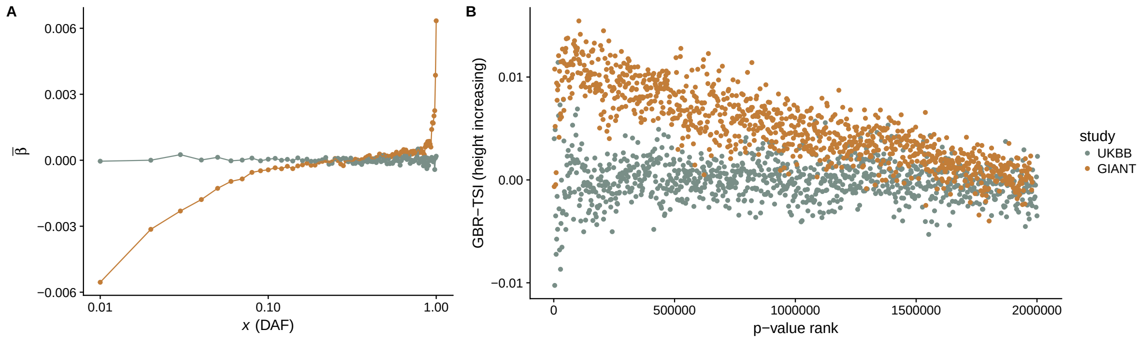

Figure S6: Correlation between mean effect size and allele frequency (A) and correlation the difference in frequency between northern and southern Europe for the height increasing allele and p-value rank (B) in the GIANT study (gold) and the UK Biobank (gray). Note that alleles were grouped in to bins of 2,000 by p-value and were not thinned by linkage.

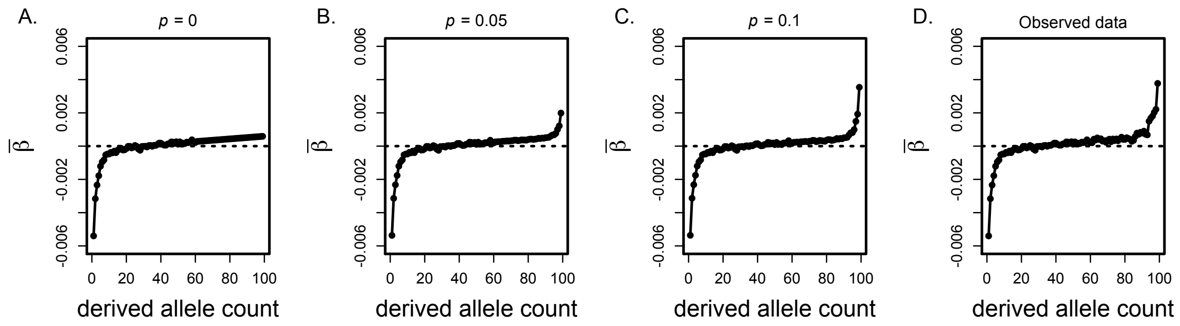

Figure S7: The impact of ancestral uncertainty on the observed value of mean β as a function of derived allele count x. Panel A shows results corresponding to no uncertainty ($p = 0$), in B $p = 0.05$, in C $p = 0.1$, and D shows the observed GIANT height data.

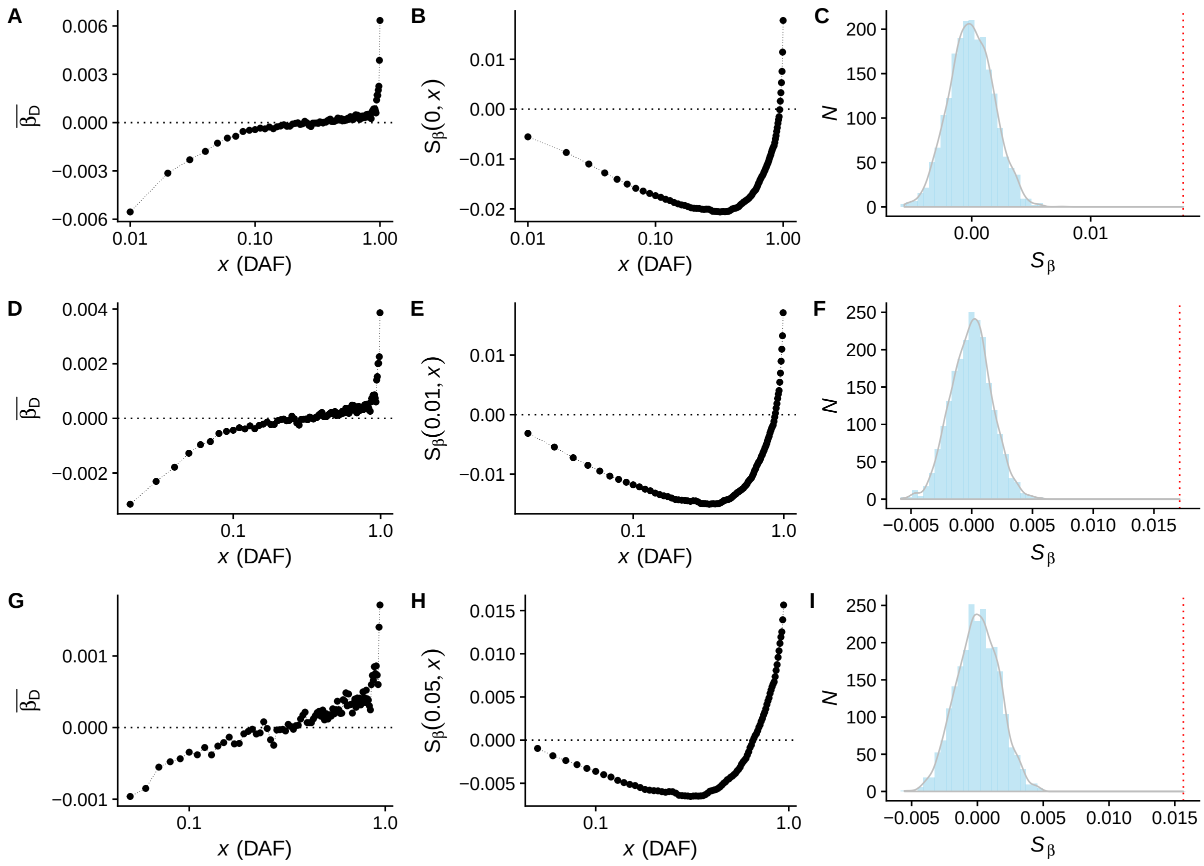

Figure S8: S_β for height. The panels in the left column show the relationship between allele frequency and $\overline{\beta}$, the middle column displays the cumulative value of $S_\beta(x_i, x_f)$, and the right columns show the null distribution of S_β given by our permutation test. Panels A-C correspond to $x_i = 0$ and $x_f = 1$, panels D-F correspond to $x_i = 0.01$ and $x_f = 0.99$, and panels G-I correspond to $x_i = 0.05$ and $x_f = 0.95$

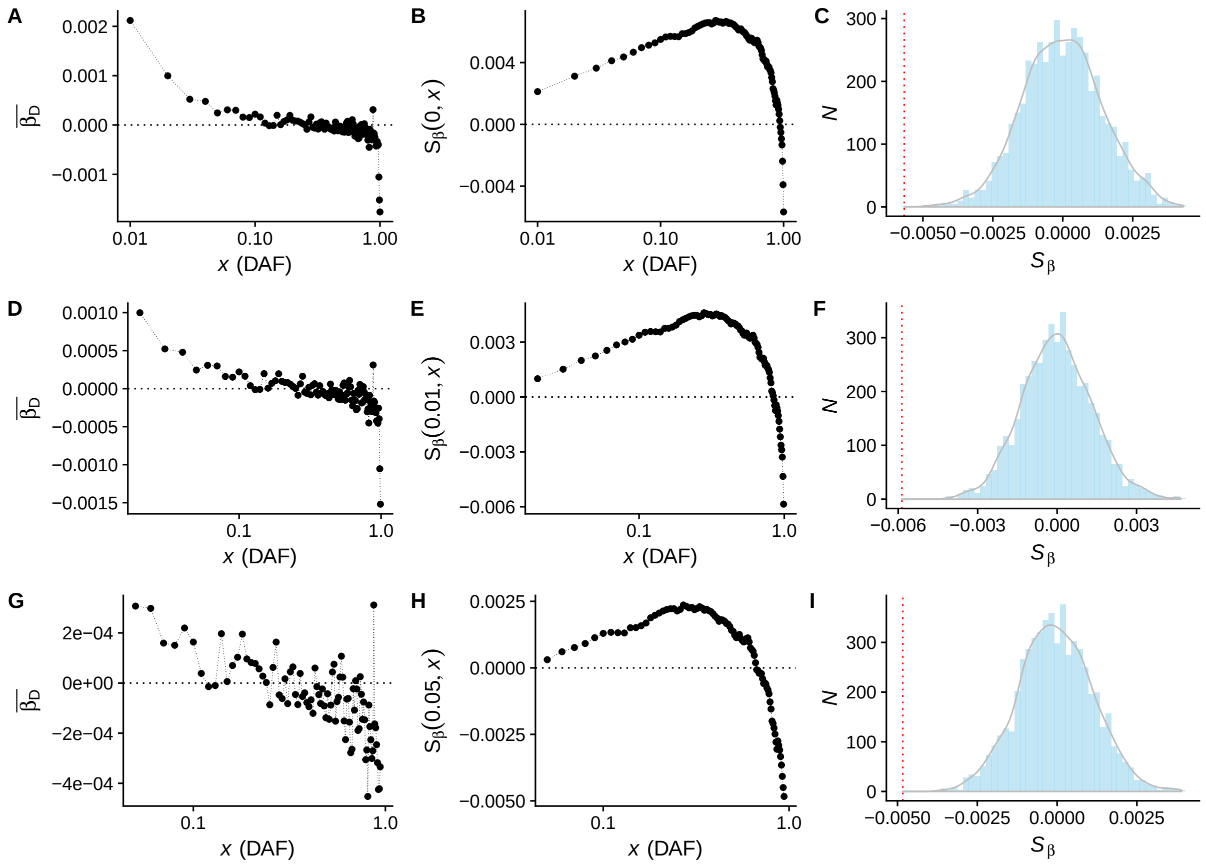

Figure S9: S_β for BMI. The panels in the left column show the relationship between allele frequency and $\overline{\beta}$, the middle column displays the cumulative value of $S_\beta(x_i, x_f)$, and the right columns show the null distribution of S_β given by our permutation test. Panels A-C correspond to $x_i = 0$ and $x_f = 1$, panels D-F correspond to $x_i = 0.01$ and $x_f = 0.99$, and panels G-I correspond to $x_i = 0.05$ and $x_f = 0.95$

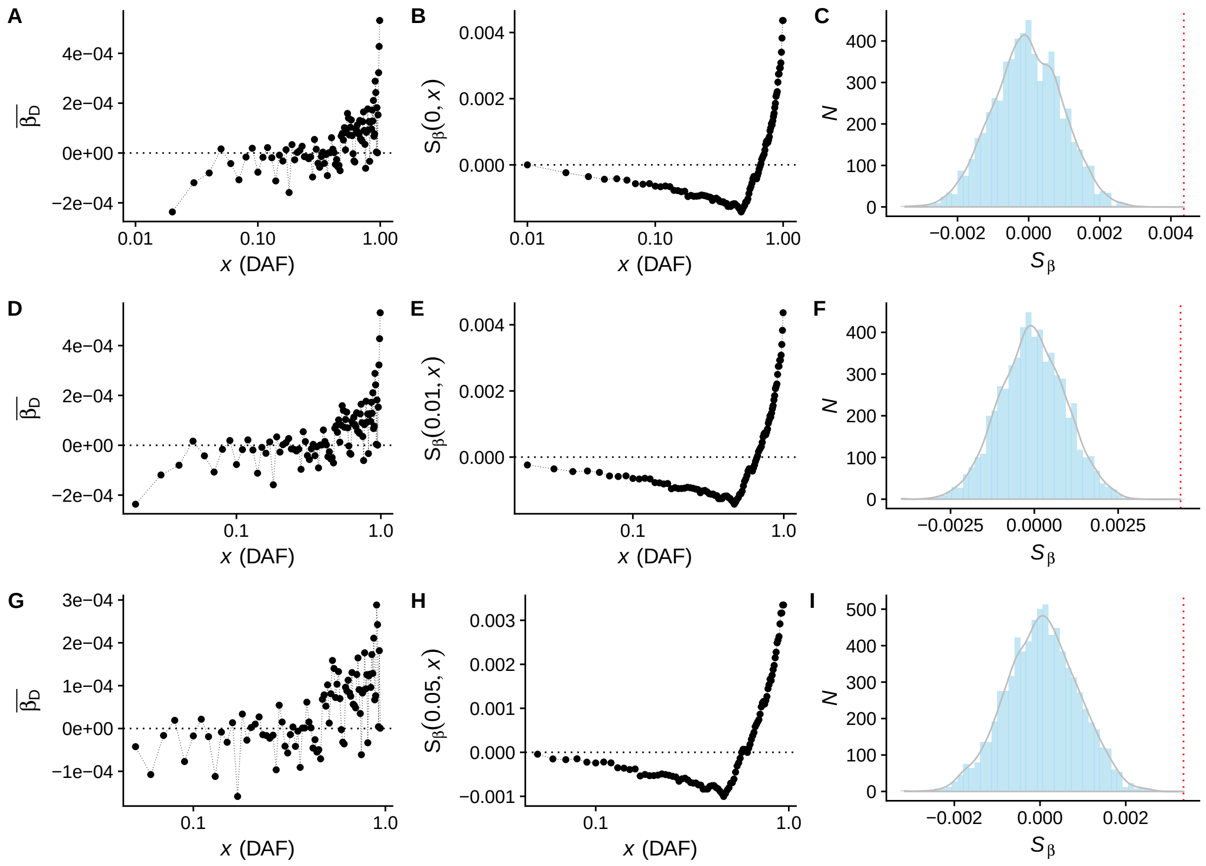

Figure S10: S_β for educational attainment. The panels in the left column show the relationship between allele frequency and $\overline{\beta}$, the middle column displays the cumulative value of $S_\beta(x_i, x_f)$, and the right columns show the null distribution of S_β given by our permutation test. Panels A-C correspond to $x_i = 0$ and $x_f = 1$, panels D-F correspond to $x_i = 0.01$ and $x_f = 0.99$, and panels G-I correspond to $x_i = 0.05$ and $x_f = 0.95$. Note that the results for A-C are the same as D-F because no alleles under 1% were included in the summary data for this study.

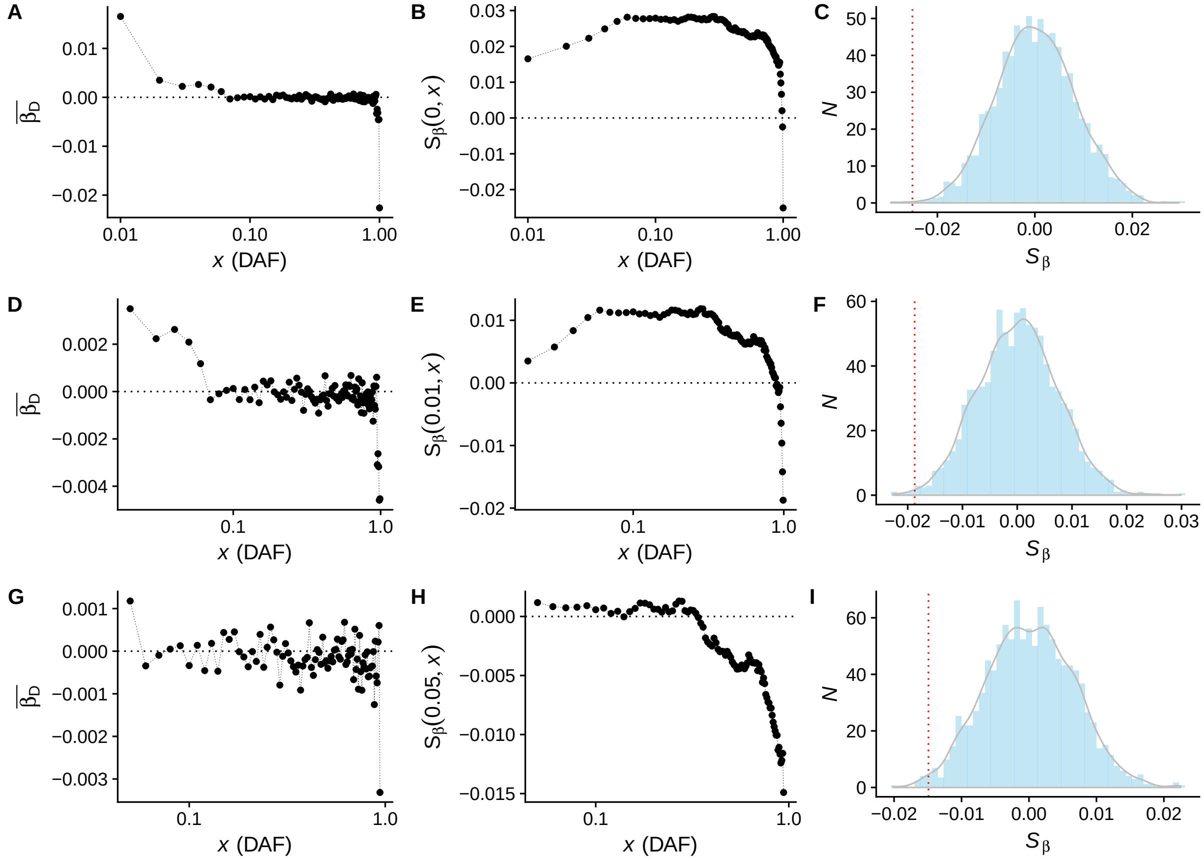

Figure S11: S_β for Crohn's disease. The panels in the left column show the relationship between allele frequency and $\overline{\beta}$, the middle column displays the cumulative value of $S_\beta(x_i, x_f)$, and the right columns show the null distribution of S_β given by our permutation test. Panels A-C correspond to $x_i = 0$ and $x_f = 1$, panels D-F correspond to $x_i = 0.01$ and $x_f = 0.99$, and panels G-I correspond to $x_i = 0.05$ and $x_f = 0.95$

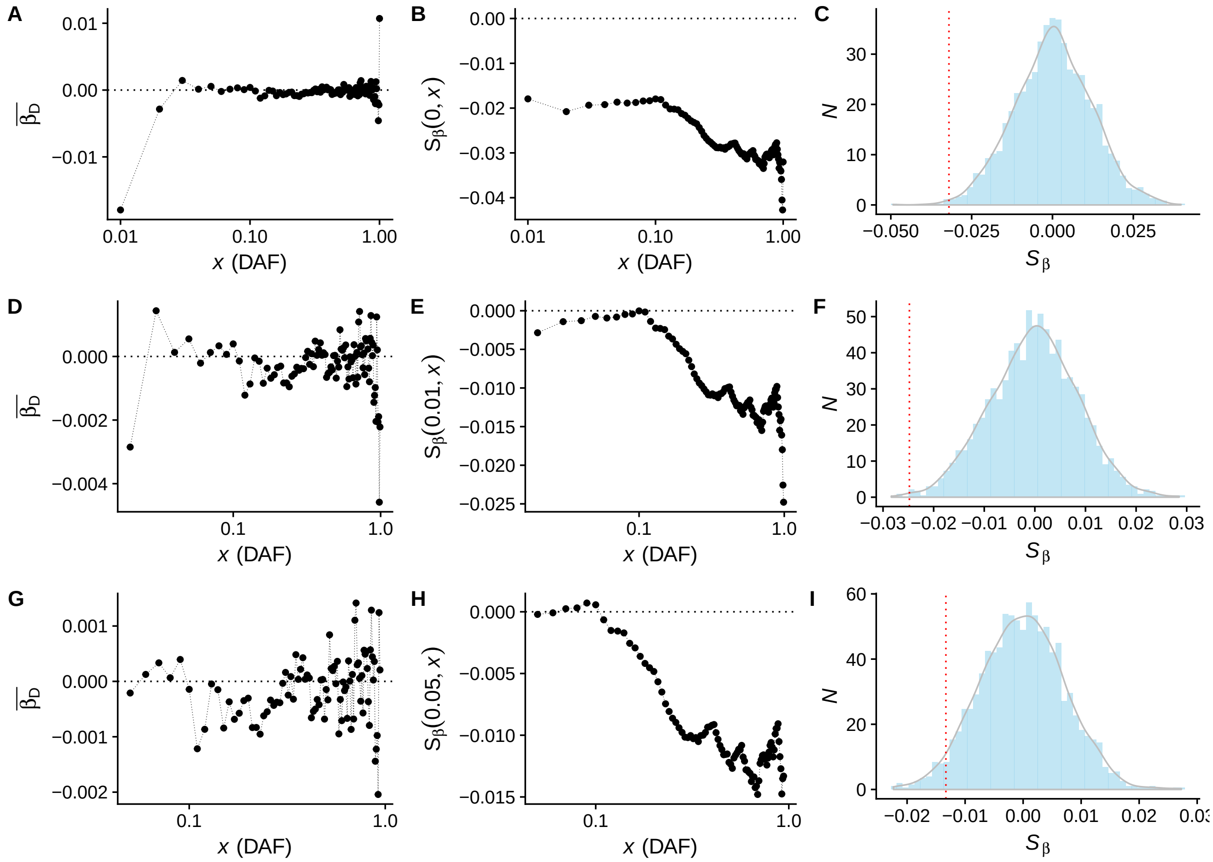

Figure S12: S_β for schizophrenia. The panels in the left column show the relationship between allele frequency and $\overline{\beta}$, the middle column displays the cumulative value of $S_\beta(x_i, x_f)$, and the right columns show the null distribution of S_β given by our permutation test. Panels A-C correspond to $x_i = 0$ and $x_f = 1$, panels D-F correspond to $x_i = 0.01$ and $x_f = 0.99$, and panels G-I correspond to $x_i = 0.05$ and $x_f = 0.95$

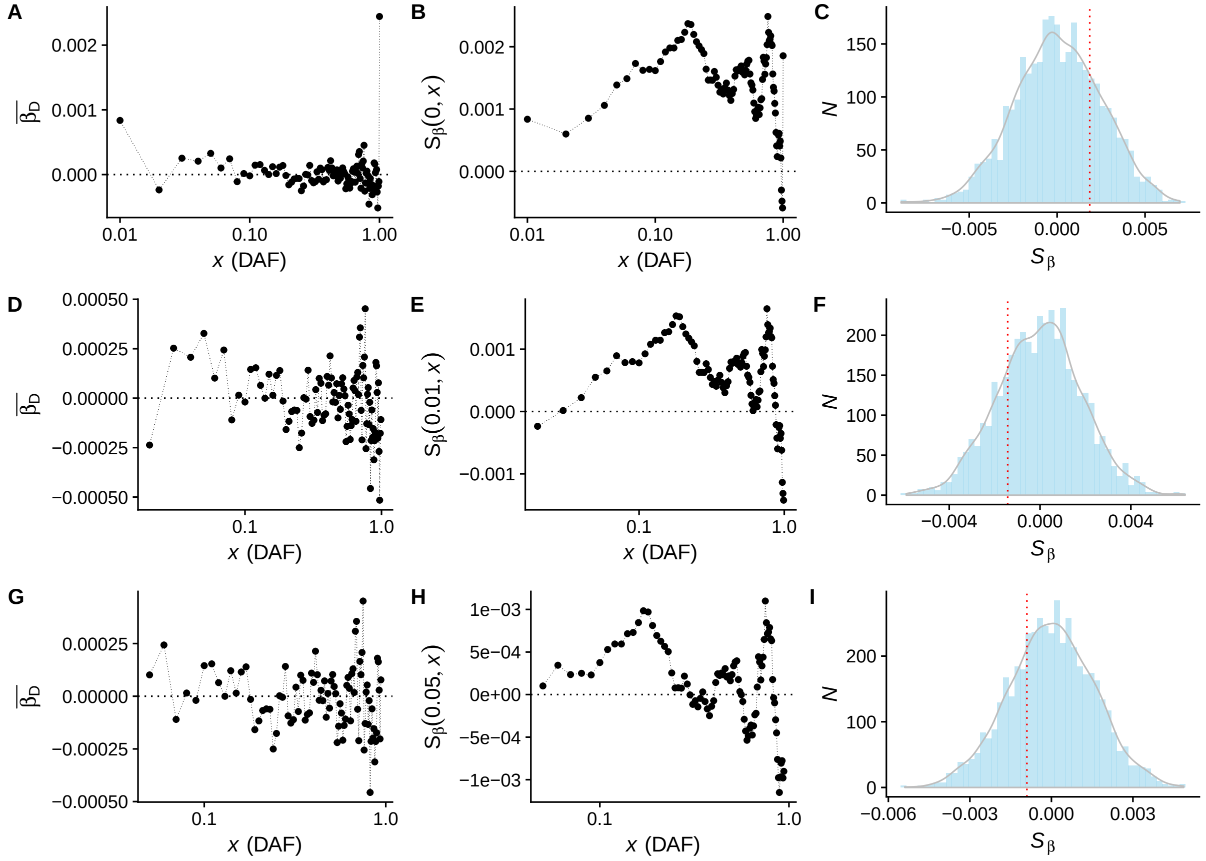

Figure S13: S_β for global lipid levels. The panels in the left column show the relationship between allele frequency and $\overline{\beta}$, the middle column displays the cumulative value of $S_\beta(x_i, x_f)$, and the right columns show the null distribution of S_β given by our permutation test. Panels A-C correspond to $x_i = 0$ and $x_f = 1$, panels D-F correspond to $x_i = 0.01$ and $x_f = 0.99$, and panels G-I correspond to $x_i = 0.05$ and $x_f = 0.95$

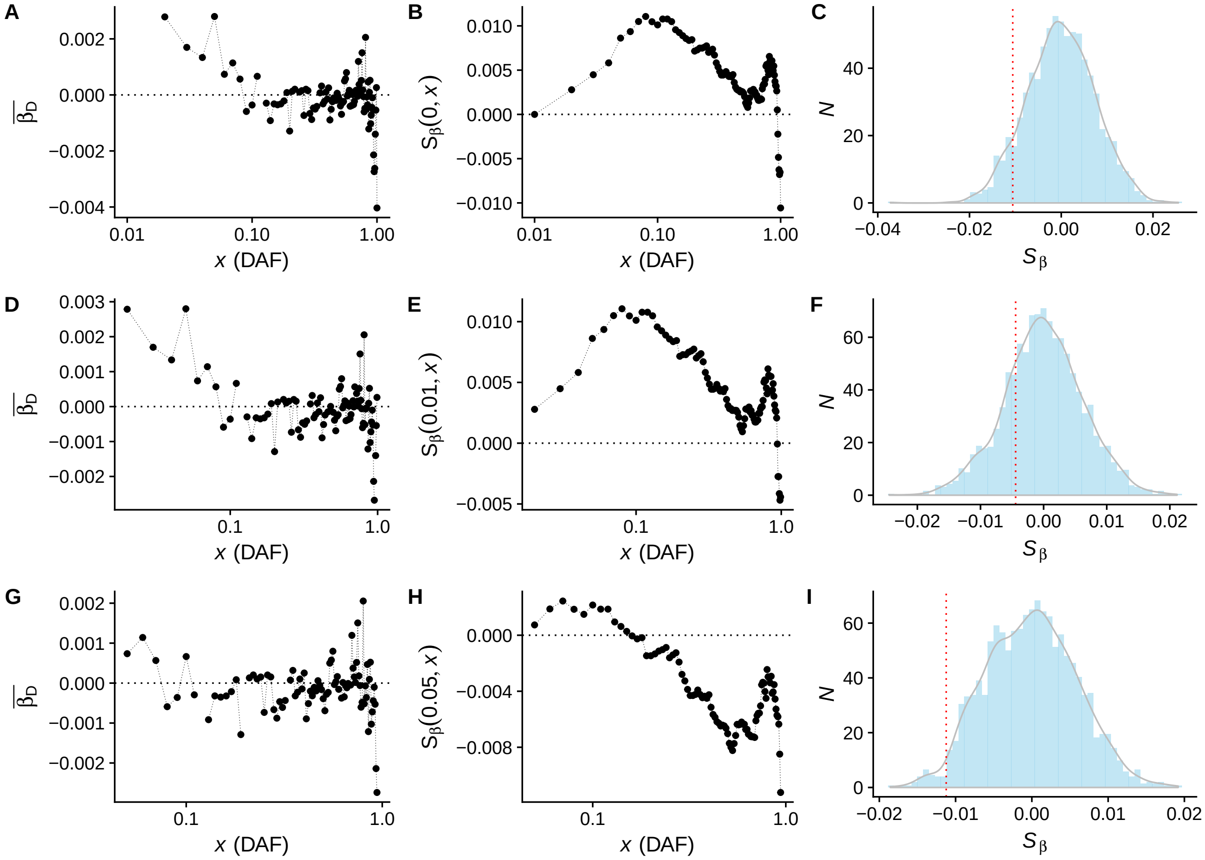

Figure S14: S_β for menopause onset. The panels in the left column show the relationship between allele frequency and $\overline{\beta}$, the middle column displays the cumulative value of $S_\beta(x_i, x_f)$, and the right columns show the null distribution of S_β given by our permutation test. Panels A-C correspond to $x_i = 0$ and $x_f = 1$, panels D-F correspond to $x_i = 0.01$ and $x_f = 0.99$, and panels G-I correspond to $x_i = 0.05$ and $x_f = 0.95$

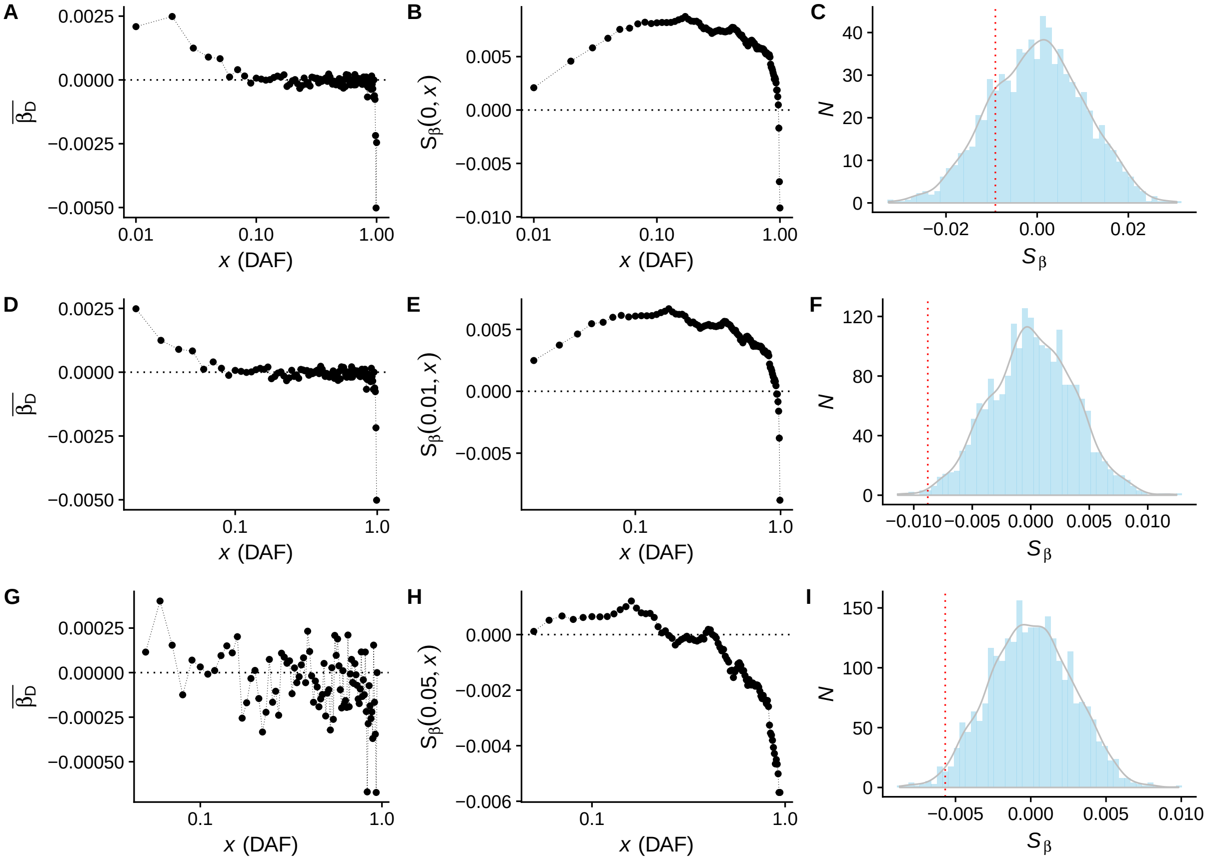

Figure S15: S_β for major depression. The panels in the left column show the relationship between allele frequency and $\overline{\beta}$, the middle column displays the cumulative value of $S_\beta(x_i, x_f)$, and the right columns show the null distribution of S_β given by our permutation test. Panels A-C correspond to $x_i = 0$ and $x_f = 1$, panels D-F correspond to $x_i = 0.01$ and $x_f = 0.99$, and panels G-I correspond to $x_i = 0.05$ and $x_f = 0.95$

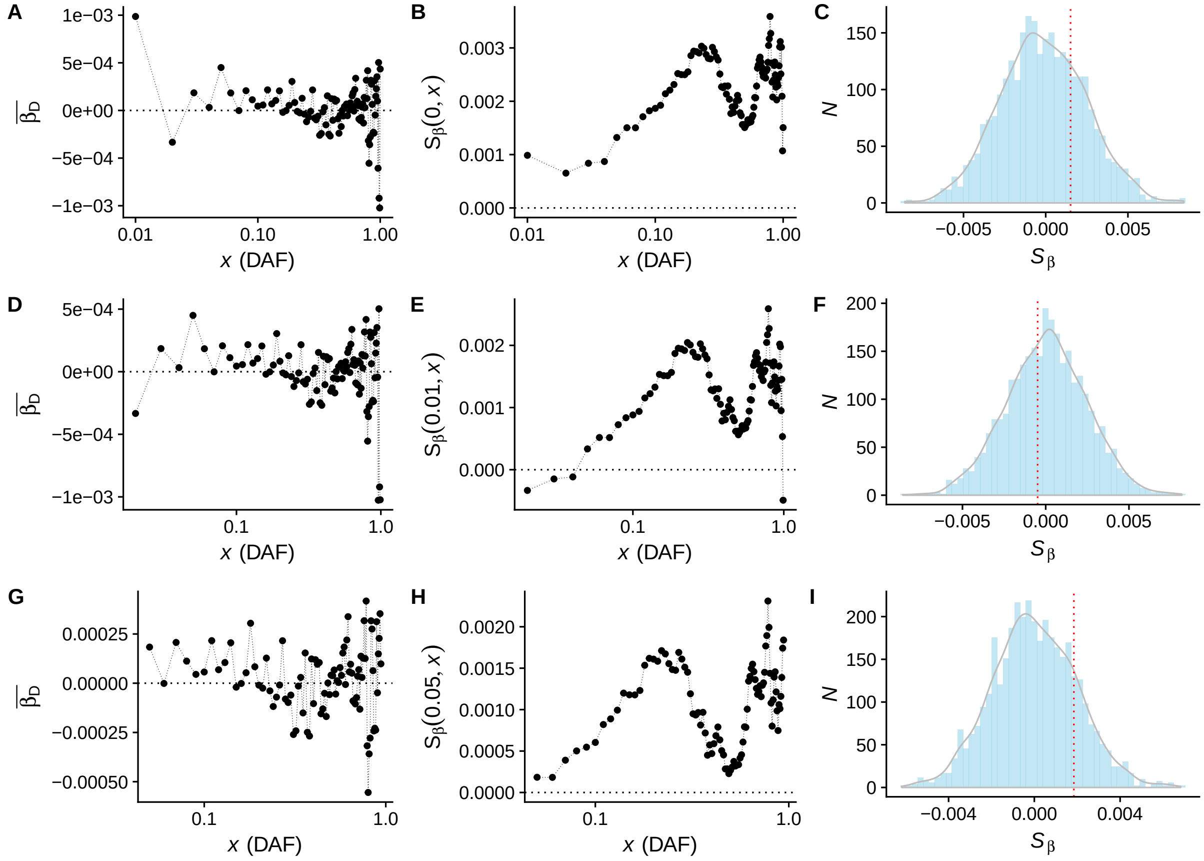

Figure S16: S_β for waist-hip ratio adjusted for BMI. The panels in the left column show the relationship between allele frequency and $\overline{\beta}$, the middle column displays the cumulative value of $S_\beta(x_i, x_f)$, and the right columns show the null distribution of S_β given by our permutation framework. Panels A-C correspond to $x_i = 0$ and $x_f = 1$, panels D-F correspond to $x_i = 0.01$ and $x_f = 0.99$, and panels G-I correspond to $x_i = 0.05$ and $x_f = 0.95$

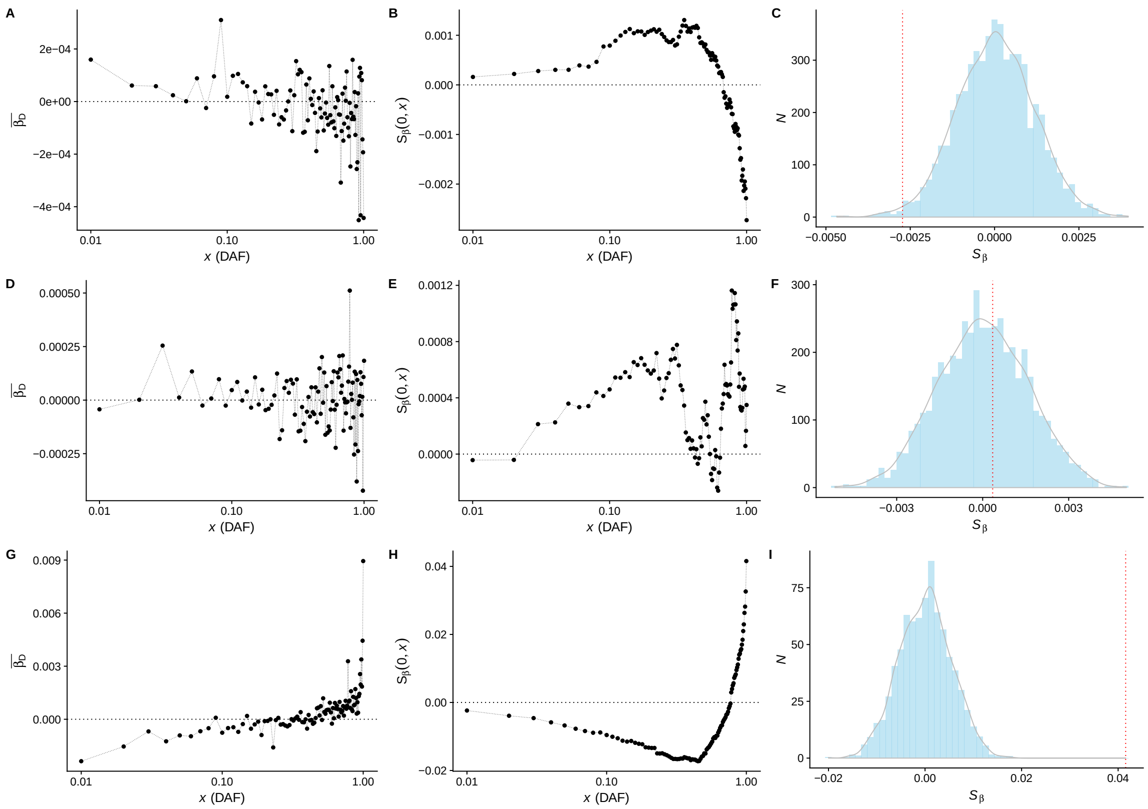

Figure S17: S_β for BMI (A-C), height (D-F), and educational attainment (G-I) in the UK Biobank. The panels in the left column show the relationship between allele frequency and $\overline{\beta}$, the middle column displays the cumulative value of $S_\beta(x_i, x_f)$, and the right columns show the null distribution of S_β given by our permutation framework.

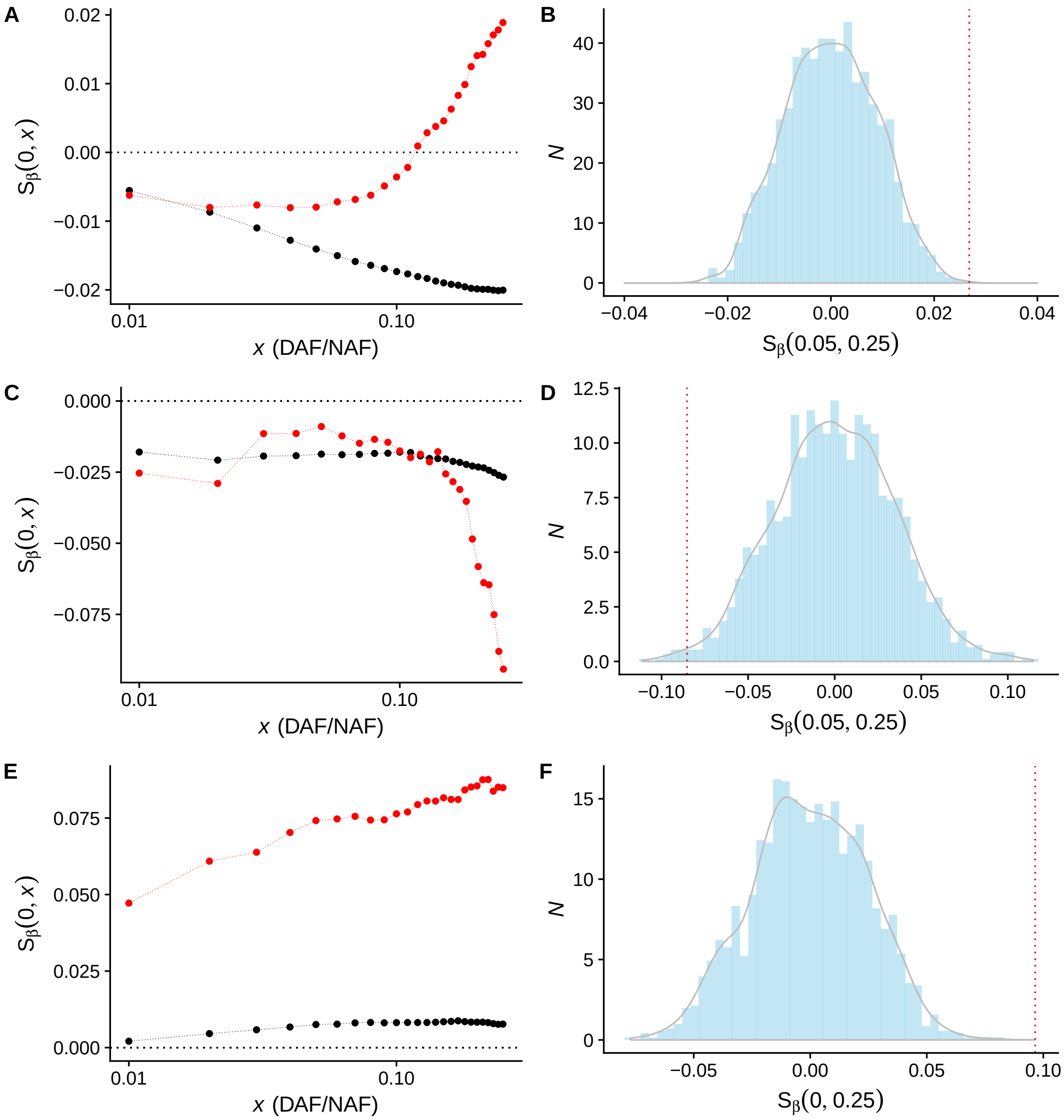

Figure S18: Signals of putative polygenic selection and mutation bias on Neanderthal alleles. A-B correspond to height, C-D correspond to schizophrenia, and E-F correspond to major depression. The left column shows $S_\beta(0, x)$ for Neanderthal alleles (red) and modern human alleles for comparison (truncated at allele frequency of 0.25). The right column shows $S_\beta(x_i, 0.25)$ for our permutations, where the observed signal is shown in the red dashed line and the value of x_i is indicated under the plot. We note that

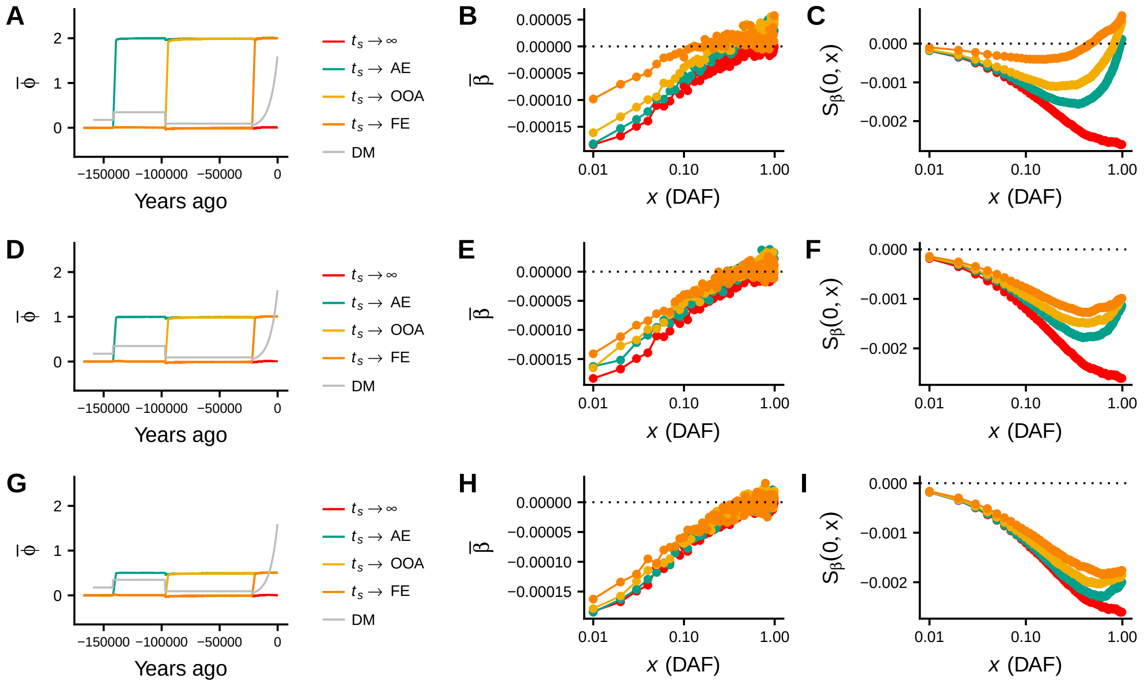

Figure S19: A-C: Simulations with the same parameters as Fig. 1C-E, but with the shift in optimal phenotype of $\Delta\phi = 2$ occurring linearly over 100 generations (2500 years), rather than instantaneously in a single generation. D-F: Simulations with the same parameters as Fig. 1C-E, but with a shift of $\Delta\phi = 1$ occurring linearly over 100 generations. G-I: Simulations with the same parameters as Fig. 1C-E, but with a shift of $\Delta\phi = 0.5$ occurring linearly over 100 generations.
